# Long Telomeres Produced by Telomerase-Resistant Recombination Are Established from a Single Source and Are Subject to Extreme Sequence Scrambling

**DOI:** 10.1371/journal.pgen.1003017

**Published:** 2012-11-01

**Authors:** Jianing Xu, Michael J. McEachern

**Affiliations:** Department of Genetics, Fred Davision Life Science Complex, University of Georgia, Athens, Georgia, United States of America; Chinese Academy of Sciences, China

## Abstract

Considerable evidence now supports the idea that the moderate telomere lengthening produced by recombinational telomere elongation (RTE) in a *Kluyveromyces lactis* telomerase deletion mutant occurs through a roll-and-spread mechanism. However, it is unclear whether this mechanism can account for other forms of RTE that produce much longer telomeres such as are seen in human alternative lengthening of telomere (ALT) cells or in the telomerase-resistant type IIR “runaway” RTE such as occurs in the *K. lactis stn1-M1* mutant. In this study we have used mutationally tagged telomeres to examine the mechanism of RTE in an *stn1-M1* mutant both with and without telomerase. Our results suggest that the establishment stage of the mutant state in newly generated *stn1-M1 ter1-Δ* mutants surprisingly involves a first stage of sudden telomere shortening. Our data also show that, as predicted by the roll-and-spread mechanism, all lengthened telomeres in a newly established mutant cell commonly emerge from a single telomere source. However, in sharp contrast to the RTE of telomerase deletion survivors, we show that the RTE of *stn1-M1 ter1-Δ* cells produces telomeres whose sequences undergo continuous intense scrambling via recombination. While telomerase was not necessary for the long telomeres in *stn1-M1* cells, its presence during their establishment was seen to interfere with the amplification of repeats via recombination, a result consistent with telomerase retaining its ability to add repeats during active RTE. Finally, we observed that the presence of active mismatch repair or telomerase had important influences on telomeric amplification and/or instability.

## Author Summary

Indefinite growth of tumor cells requires a mechanism to maintain telomeres. While most cancers use telomerase for this, some maintain long and heterogeneous telomeres using a recombination-dependent mechanism termed alternative lengthening of telomeres (ALT). What causes ALT and how their long and heterogeneous telomeres form and are maintained are not well understood. In this study, we use mutationally tagged telomeric repeats to probe the mechanisms by which highly elongated telomeres are generated by recombination in an ALT–like yeast mutant. Our data show that most or all lengthened telomeres in a newly established mutant cell are commonly generated by amplifying sequence from a single telomere source. This is consistent with the roll-and-spread model, which proposes that a single circle of telomeric DNA can be the ultimate source of all newly amplified telomeres. Other evidence showed that the telomeres of the mutant are exceptionally dynamic. Rapid terminal deletions preceded telomere elongation at the establishment of the mutant state. Also, patterns of telomeric repeats present in long telomeres became rapidly scrambled. These findings may have implications for the establishment and maintenance of long telomeres in human ALT cells.

## Introduction

Recombination can maintain telomeres in many situations where telomerase is absent. Natural examples of this include the chromosomal telomeres in the mosquito Anopheles [1]–[2] and the mitochondrial telomeres in certain ciliates and yeasts [3]–[5]. Of particular importance are the 5–10% of human cancer cells where telomerase activity is undetectable and telomeres are maintained by a mechanism termed Alternative Lengthening of Telomeres (ALT) (for a review, see [6]). ALT cells are characterized by long and heterogeneous telomeres [7]–[10] and the presence of ALT-associated PML bodies (APB) that contain telomeric DNA as well as telomeric and recombinational proteins [8], [11]–[14].

Several lines of evidence suggest that recombination is involved in maintaining telomeres when the long and heterogeneous telomeres already exist in ALT cells. Plasmid tags introduced into a telomere can be duplicated to other telomeres or at the same telomere in ALT cells but not in telomerase positive cells [15]–[16]. Extrachromosomal telomeric circles (t-circles), likely products of intratelomeric recombination, are abundant in ALT cells [17]–[19]. Telomeric sister chromatid exchanges (t-SCE) occur at highly elevated rates in ALT cells [20]–[22]. However, the details of how recombination can establish these long and heterogeneous telomeres from normal-length telomeres in ALT cells are still unknown.

Recombinational telomere elongation (RTE) has been described in yeast mutants lacking telomerase in the species *Sacchromyces cerevisiae* [23], *Kluyveromyces lactis* [24], *Candida albicans* [25] and *Schizosacchromyces pombe* [26]. The recombination in these cases is thought to be caused by the shortening telomeres eventually losing part or all of their protective capping function. These mutants commonly display gradual growth senescence when telomeres are gradually shortening that is followed by the formation of better growing post-senescence survivors with longer telomeres [23]–[24], [27]. Two types of RTE were initially described in telomerase deletion mutants of *S. cerevisiae*. Both depend upon *RAD52*, suggesting that they require homologous recombination (HR). Type I RTE is characterized by amplification of subtelomeric Y′ elements and short tracts of telomeric repeats, and is dependent upon the canonical mitotic HR pathway involving *RAD51*, *RAD54*, *RAD55*, and *RAD57*. Type II RTE is characterized by lengthened tracts of telomeric repeats and is dependent upon a different pathway involving *RAD50*, *RAD59*, and *SGS1* [28]–[33]. Only type II RTE normally occurs in *K. lactis* telomerase deletion mutants (*ter1-Δ*) [24]. Studies, particularly in *K. lactis*, have suggested that type II RTE occurs through a roll-and-spread mechanism, where a t-circle is used as a template to lengthen one short telomere which in turn can be used as a template to lengthen other telomeres via break-induced replication (BIR) events [34]–[37]. Consistent with this model, post-senescence survivors derived from cells with two kinds of telomeric repeats often contain repeating patterns in most or all lengthened telomeres [36]. Additionally, when a DNA circle containing telomeric repeats is transformed into a *K. lactis* telomerase deletion mutant, its sequence becomes efficiently amplified onto telomeric ends as long tandem arrays [36]. T-circles are also abundant in yeast mutants with telomere dysfunction [34], [38]–[39]. Furthermore, sequence from a single telomere is used as the source of all lengthened telomeres in *K. lactis* post-senescence survivors [37]. Type II RTE in *S. cerevisiae* has also been suggested to involve rolling circle copying of t-circles [39].

RTE can also be triggered by perturbation of telomeric capping proteins. For example, in *S. cerevisiae*, a *cdc13-1 yku70* mutant can generate type II survivors without gradual growth senescence [40]. In *K. lactis*, telomerase deletion mutants containing telomeric repeats with defects in Rap1 binding develop much longer telomeres than equivalent mutants with only wild type repeats [37], [41]. Of particular interest is the *stn1-M1* mutant of *K. lactis* [42]. Stn1 is a part of the Cdc13/Stn1/Ten1 (CST) complex that binds to the 3′ single-stranded telomeric overhang and protects the telomere termini from degradation and engagement in recombination (for a review see [43]). Stn1 also regulates telomerase recruitment and telomeric C-strand synthesis, the latter via its interaction with Polα/primase [44]–[45]. In many ways, the *stn1-M1* mutant displays more similarity to ALT than do *ter1-Δ* mutants. It shares with ALT a steady state of very long and highly heterogeneous telomeres that are produced by recombination as well as the immediate presence of chronic but slight growth defects instead of the gradual growth senescence and survivor formation seen in *ter1-Δ* mutants [8], [42], [46]. Both ALT cells and *stn1-M1* cells show high levels of telomere instability including elevated telomere recombination, rapid telomere deletions, and abundant extrachromosomal telomeric DNA including t-circles [10], [21], [38], [42], [47]–[48]. Finally, the phenotypes of *stn1-M1* and of most ALT cells, in sharp contrast to that of *ter1-Δ* mutants, are largely not affected by whether telomerase is present or not, a property that we refer to as telomerase-resistant [8], [42]. While telomeric recombination in *ter1-Δ* cells appears repressed once telomeres are even moderately elongated, the telomere recombination in *stn1-M1* cells is thought to occur at telomeres of all sizes. To distinguish the fundamental differences between telomere capping defects in the two mutants, the RTE in *stn1-M1* mutant was termed type IIR for its “runaway” lengthening characteristics [42]. Given its similarities with ALT cells, the *stn1-M1* mutant may therefore be a useful model system to obtain more clues about mechanisms that establish long telomeres in ALT cells.

In this work, we utilize mutationally tagged telomeric repeats to study the mechanism of type IIR RTE in *stn1-M1* mutant during the establishment stage where long telomeres are generated from much shorter telomeres. Our results are consistent with predictions of the roll-and-spread model in demonstrating that sequence from one telomere is commonly spread to most or all telomeres of newly formed *stn1-M1* mutants. Our results also suggest that rapid telomere truncations routinely precede the generation of long telomeres and that the presence of active mismatch repair or telomerase can impact the outcomes observed.

## Results

### Generating *stn1-M1* mutants from precursors with mutationally tagged telomeric termini

**Figure 1:**
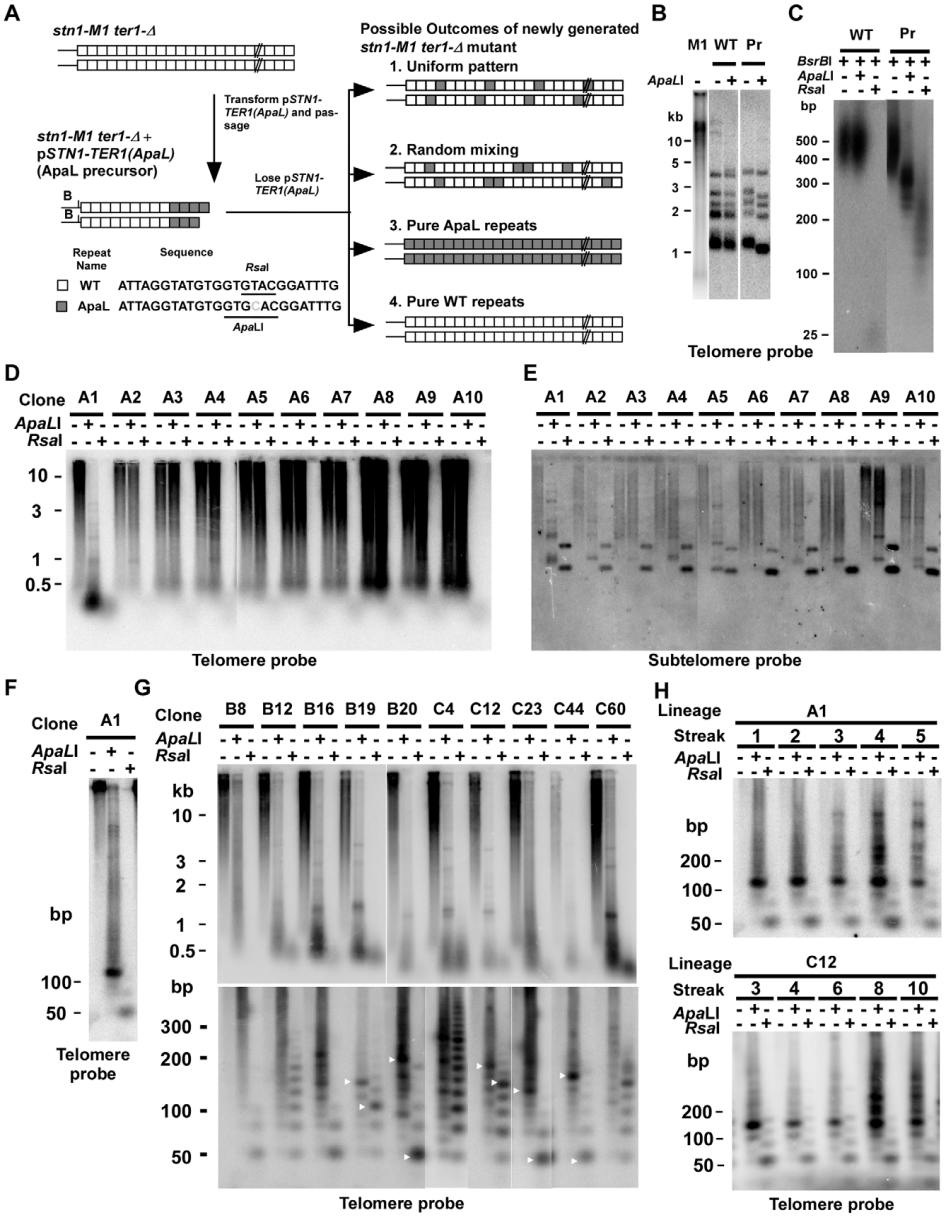
Use of mutationally tagged repeats at telomeric termini to study RTE in *stn1-M1* cells reveals evidence for repeating structures in telomeres of some newly generated *stn1-M1 ter1-Δ* mutants. (A) Experiment outline. After transformation of p*STN1-TER1(ApaL)* into *stn1-M1 ter1-Δ* strains, followed by serial passaging for 10 streaks, the ApaL precursor was generated that contains telomeres composed of terminal ApaL repeats (gray boxes) and internal WT repeats (white boxes). The sequences of a WT repeat and an ApaL repeat are shown. The positions of *Apa*LI and *Rsa*I restriction sites in the repeats and the subtelomeric *Bsr*BI(B) site are indicated. Upon loss of p*STN1-TER1(ApaL),* newly generated *stn1-M1 ter1-Δ* mutants with long telomeres can be recovered. Four possible outcomes for the telomeres in these mutants are illustrated; 1. A uniformly repeating pattern of the two repeat types, 2. Randomly mixed repeats, 3. Pure ApaL repeats and 4. Pure WT repeats. (B) Southern blot, hybridized with a telomere probe, of *Eco*RI (indicated by “−”) and *Eco*RI+*Apa*LI (indicated by “+”) digested DNA from *stn1-M1* (M1), WT and ApaL precursor (Pr). (C), *Bsr*BI, *Bsr*BI+*Apa*LI and *Bsr*BI+*Rsa*I-digested DNA from WT and ApaL precursor (Pr) separated on a 3% agarose gel electrophoresis and hybridized with the telomere probe. (D) Southern blot, hybridized with a telomere probe, of *Eco*RI (1^st^ lane of every clone), *Eco*RI+*Apa*LI (2^nd^ lane of every clone) and *Eco*RI+*Rsa*I (3^rd^ lane of every clone) digested DNA from 10 newly generated *stn1-M1 ter1-Δ* mutants generated from the ApaL precursor. (E) Same filter as in panel D after stripping and rehybridization with a subtelomeric probe. (F) *Eco*RI (1^st^ lane), *Eco*RI+*Apa*LI (2^nd^ lane) and *Eco*RI+*Rsa*I (3^rd^ lane) digested DNA from A1 clone in panel A separated on a 3% agarose gel and hybridized with a telomere probe. (G) Southern blot, hybridized with a telomere probe, of *Eco*RI (1^st^ lane of every clone), *Eco*RI+*Apa*LI (2^nd^ lane of every clone) and *Eco*RI+*Rsa*I (3^rd^ lane of every clone) digested DNA from 10 additional newly generated *stn1-M1 ter1-Δ* mutants separated by electrophoreses on 0.8% (Upper panel) and a 3% (Bottom panel) agarose gels. (H) Southern blot, hybridized with a telomere probe, of *Eco*RI (1^st^ lane of every clone), *Eco*RI+*Apa*LI (2^nd^ lane of every clone) and *Eco*RI+*Rsa*I (3^rd^ lane of every clone) digested DNA from A1 in panel D and C12 in panel G passaged for multiple streaks (as indicated) on YPD medium.

To study the type IIR RTE that forms highly elongated telomeres in the *stn1-M1* mutant of *K. lactis*, we generated *stn1-M1* mutants from two kinds of precursors with mutationally tagged telomeric repeats. Previously, similar approaches were informative in studying the type II RTE that forms the more modestly elongated telomeres in *K. lactis* telomerase deletion (*ter1-Δ*) mutants [36]–[37]. The experimental setup for generating *stn1-M1* cells from the first kind of precursor is diagramed in Figure 1A. An *stn1-M1 ter1-Δ* mutant was first transformed with a plasmid (p*STN1*-*TER1(ApaL)*) containing both the *STN1* and the *TER1-20C(ApaL)* genes. The *TER1-20C(ApaL)* gene forms a telomerase that adds mutated ApaL repeats onto all telomeric termini. ApaL repeats are phenotypically silent but contain a single base change that both forms an *Apa*LI site and eliminates the native *Rsa*I site [49] (Figure 1A). A transformant was then serially passaged for ten streaks to allow telomeres to shorten to near normal length and to incorporate ApaL repeats at their termini. These passaged cells are referred to as the ApaL precursor cells.

All telomeres in the ApaL precursor cells had acquired ApaL repeats at termini as indicated by their *Eco*RI-digested telomeric fragments being shorter after *Apa*LI digestion (Figure 1B and data not shown). In contrast, telomeric fragments in a wild type control were digested away with *Rsa*I but resistant to *Apa*LI (Figure 1B–1C). To estimate the number of ApaL repeats at telomeric termini of ApaL precursors, we digested their DNA by *Bsr*BI+*Apa*LI and *Bsr*BI+*Rsa*I (Figure 1C and data not shown). *Bsr*BI cleaves 3 bp away from 10 of 12 telomeres in *K. lactis* (Figure 1A), and *Apa*LI and *Rsa*I specifically cleave ApaL and wild type (WT) repeats, respectively. The size ranges of the telomeric signal in *Bsr*BI+*Apa*LI and *Bsr*BI+*Rsa*I digestions reflect the size ranges of internal WT repeats and terminal ApaL repeats, respectively. From this, we estimated that the terminal 100–300 bp of the 350–600 bp total telomere length was composed of ApaL repeats. This was verified by cloning and sequencing two telomeres from an ApaL precursor. One cloned telomere contained 11 basal WT repeats and three terminal ApaL repeats and the other contained ∼9 basal WT repeats and five terminal ApaL repeats (Figure S1A). To our surprise, clone 2 also contained a 13 bp repeat located between the WT and ApaL repeats (Figure S1A). The sequence of this 13 bp repeat suggested it arose when the terminal bases of a telomere annealed to the middle instead of to the 3′ end of the telomerase RNA template (Figure S1B). This half-sized repeat was not likely formed by the ApaL telomerase because it contained the native *Rsa*I site of WT repeats rather than an *Apa*LI site.

We then selected for newly generated *stn1-M1 ter1-Δ* mutants by plating the ApaL precursors on medium containing 5-flouro-orotic acid (5-FOA) to select for loss of the p*STN1*-*TER1(ApaL)* plasmid. As the loss of plasmid sequences simultaneously deletes both telomerase and the wild type *STN1* gene, the generation of long telomeres in the newly formed *stn1-M1 ter1-Δ* cells should depend solely on recombination. Although the *ter1-Δ* mutation is, by itself capable of causing RTE, it is not likely to interfere with the type IIR RTE brought on by the *stn1-M1* mutation. This is because the telomeric recombination induced by the *ter1-Δ* mutation is confined to occurring when telomeres become <∼150 bp in length [37] while telomeric recombination in *stn1-M1* occurs even at very long telomeres. Consistent with this, the growth and telomere phenotypes of the *stn1-M1* mutant are epistatic to those of the *ter1-Δ* mutant.

Because the p*STN1*-*TER1(ApaL)* plasmid became integrated into a chromosome at the *stn1-M1* locus in the ApaL precursor during passaging (data not shown), the rate of recovering *stn1-M1 ter1-Δ* mutants (generated by homologous recombination looping out the plasmid from the chromosome) was relatively low. A group of ten newly generated *stn1-M1 ter1-Δ* mutants was initially analyzed. All ten mutant clones showed the long and heterogeneous pattern of telomeres that is the characteristic of the *stn1-M1* phenotype when *Eco*RI digests were observed in a Southern blot (Figure 1D). Telomeric signals in nine of these ten mutants (clones A2–A10, Figure 1D) were not obviously cleaved by *Apa*LI. Hybridization of the same filter to a subtelomeric probe (Figure 1E) showed that most signal from most clones was unchanged by *Apa*LI digestion. These results indicate that telomeres in these nine mutants were composed almost entirely of WT repeats. Consistent with this interpretation, telomeric signals from these nine mutants were virtually cleaved away by *Rsa*I, which specifically cleaves WT repeats (Figure 1D). These results are very surprising, because the *stn1-M1* phenotype forms rapidly without a period of growth senescence and should therefore not undergo any gradual sequence loss of terminal ApaL repeats before the formation of long telomeres by RTE [42]. Each of the A2–A10 clones did show a small percentage of the heterogeneously smeared subtelomeric signal shifted down to one or more bands, generally of ∼1 kb, from *Apa*LI digestion. This indicates that one or a small number of the telomeres in each clones retained at least one ApaL repeat near their base.

Strikingly, the telomeric signal of one *stn1-M1 ter1-Δ* mutant (clone A1) was cleaved into very small fragments by both *Apa*LI and *Rsa*I (Figure 1D). When the same digests were run on a high-percentage agarose gel, the small fragments were observed to be composed largely of a ∼125 bp band in the *Apa*LI digests and a ∼50 bp band in the *Rsa*I digests (Figure 1F). The former was predicted to contain four WT repeats and two half ApaL repeats and the latter was predicted to contain one ApaL repeat and two half WT repeats. These data suggest that telomeres in this *stn1-M1 ter1-Δ* mutant may contain repeating structures that consist of four WT repeats and one ApaL repeat as the repeating unit. To test this, we cloned and sequenced 38 telomeric fragments from this clone, which were produced by partial *Apa*LI digestion. Although mostly very small *Apa*LI fragments were recovered (Figure S2), the results showed that 19 of 51 (37%) blocks of WT repeats were ∼100–125 bp, of which 15 (79%) consisted of three WT repeats and one half WT repeat of the same sequence that was recovered from the ApaL precursor. Although these results rule out the presence of perfectly repeating patterns when DNA was isolated from the A1 mutant, the widespread presence of a particular pattern of repeats could suggest that telomeres with more perfect repeating patterns originally existed but was disrupted by numerous later recombination events.

We next analyzed 83 additional *stn1-M1 ter1-Δ* mutants generated from ApaL precursors. 73 of these clones had telomeric signals that were essentially uncleaved by *Apa*LI but were nearly fully cleaved by *Rsa*I indicating that the lengthened telomeres were composed of virtually all WT repeats (data not shown). However, ten clones had telomeric signals that were cleaved partly or entirely into short fragments by both *Apa*LI and *Rsa*I digestions (Figure 1G, upper panel). The same digests of these ten mutants were then run on a high-percentage agarose gel to resolve short DNA fragments (Figure 1G, lower panel). Several of these mutants, including B19, B20, C12, C23 and C44 showed favored fragment sizes in both *Apa*LI and *Rsa*I digests (indicated by white arrows) which could be indicative of degraded repeating patterns. In each of these mutants, the most common fragment size of ApaL repeats was smaller than that of WT repeats. The other mutants examined, B8, B12, B16, C4 and C60, sometimes exhibited favored short fragments in *Rsa*I digests but not obviously any in *Apa*LI digests. The average size of the telomeric signal in *Apa*LI digests of these clones tended to migrate at greater average size than that seen in the other clones. In some clones, most notably C4, the *Apa*LI digestion produced ladders of bands that included sizes consistent with the presence of the “half” telomeric repeats as was present in the A1 clone.

Two newly generated *stn1-M1 ter1-Δ* mutants that exhibited the best evidence of repeating patterns (clones A1 and C12) were serially passaged for 5–10 streaks on YPD plates and periodically examined for their telomeric DNA structure by Southern blots of *Apa*LI and *Rsa*I digests run on high-percentage agarose gels (Figure 1H). The initial banding pattern of these mutants became more complicated after passaging. Specifically, the favored fragments of the two mutants in *Apa*LI digests, initially ∼110–150 bp, became more variable at later streaks and tended to produce new fragments of larger sizes. This observation supports the idea that telomeres in *stn1-M1* mutants are extremely dynamic and are prone to high rates of recombination that can rapidly alter their structure.

### The absence of mismatch repair leads to more heterogeneously structured telomeres in newly made *stn1-M1* mutants

**Figure 2:**
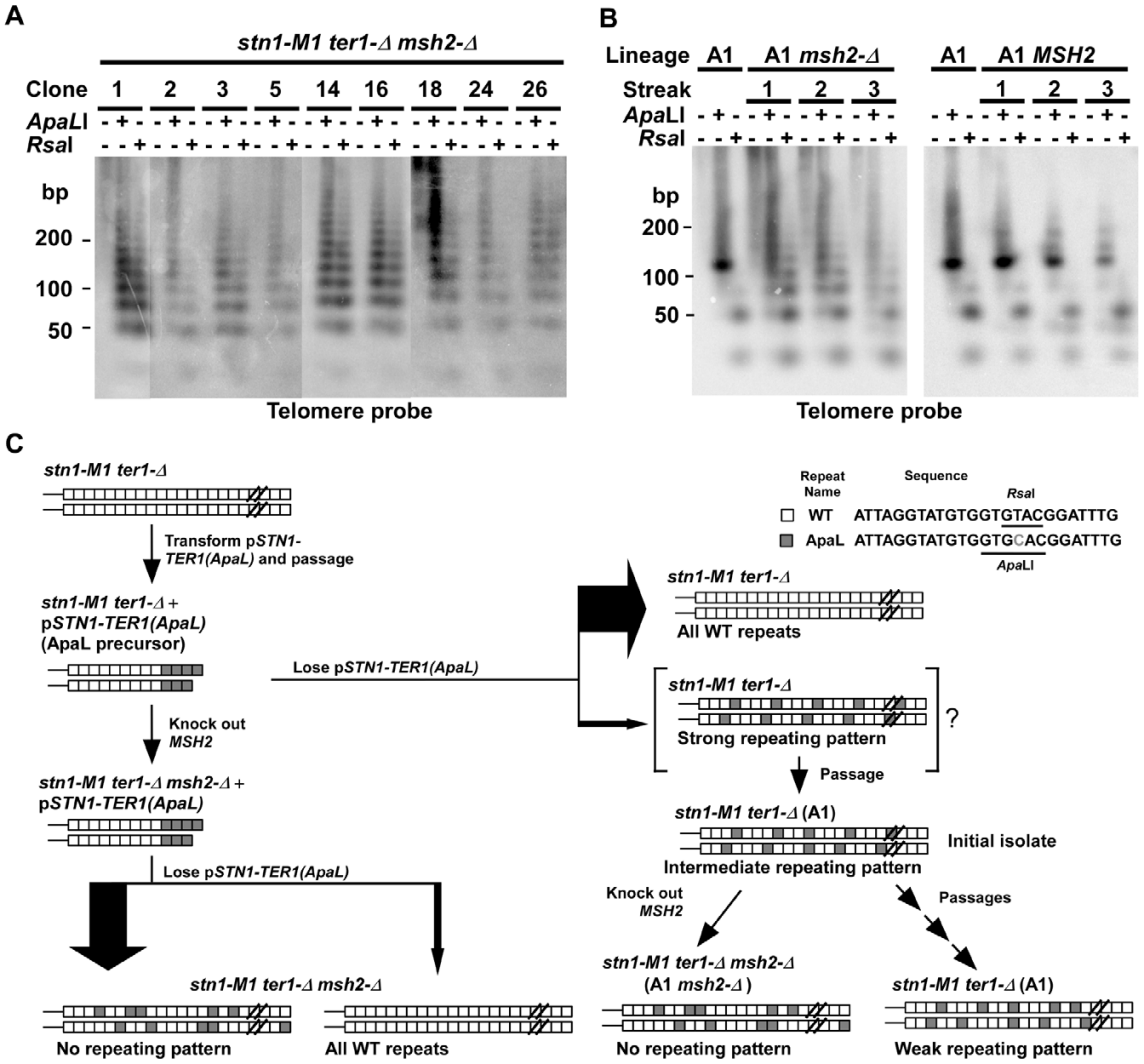
The effect of mismatch repair deficiency on telomeric structure in *stn1-M1* mutants derived from the ApaL precursor. (A) Southern blot, hybridized with a telomere probe, of *Eco*RI (1^st^ lane of every clone), *Eco*RI+*Apa*LI (2^nd^ lane of every clone) and *Eco*RI+*Rsa*I (3^rd^ lane of every clone) digested DNA from nine newly generated *stn1-M1 ter1-Δ* mutants in a *msh2-Δ* background separated on a 3% agarose gel. (B) Southern blot, hybridized with a telomere probe, of *Eco*RI (1^st^ lane of every clone), *Eco*RI+*Apa*LI (2^nd^ lane of every clone) and *Eco*RI+*Rsa*I (3^rd^ lane of every clone) digested DNA from the A1 clone (in Figure 2A) with (A1 *msh2-Δ*) or without (A1 *MSH2*) successful deletion of *MSH2*. Each clone is shown at each of 3 serial streaks. The DNA was separated on a 3% agarose gel. The A1 clone from Figure 2A is also shown. (C) Diagram summarizing the experimental outline and results from generating *stn1-M1 ter1-Δ MSH2* and *stn1-M1 ter1-Δ msh2-Δ* mutants from ApaL precursors. Only a minority of newly generated mutants retain both wild type telomeric repeats (white boxes) and ApaL repeats (grey boxes) in their lengthened telomeres. The *stn1-M1 ter1-Δ MSH2* clones such as the A1 clone are shown as hypothetically being derived from a newly established mutant cell with long telomeres with a strongly repeating pattern of wild type and ApaL repeats (“strong repeating pattern”). By the time enough cells are available for DNA analysis, recombination is likely to have partly degraded the initial pattern (“intermediate repeating pattern”). Subsequent passaging of these cells leads to further pattern degradation (“weak repeating pattern”). In contrast, an equivalent generation of *stn1-M1 ter1-Δ msh2-Δ* mutants (left side of figure) leads to no sign of repeating patterns in cells retaining both ApaL and WT repeats, even immediately after their isolation.

Base mismatches in DNA can reduce the rate of homologous recombination [50]. Therefore, the base mismatch between WT and ApaL repeats is likely to interfere with the recombination between telomeres that contain them. To test this, we disrupted the *MSH2* gene (required for mismatch repair) in an ApaL precursor, and generated 17 *stn1-M1 ter1-Δ msh2*-*Δ* mutants by losing p*STN1*-*TER1(ApaL)* as described above. Telomeric signals in 5 of these mutants showed little or no cleavage by *Apa*LI (data not shown). However, telomeric signals in the other 12 mutants were cleaved into broad ladders of bands with ∼25 bp steps by both *Apa*LI and *Rsa*I (Figure 2A and data not shown). Unlike what was seen in mutants derived from a *MSH2* background, these mutants showed no obvious favored fragments that might have indicated the presence of a degraded pattern. The appreciably higher percentage of *stn1-M1* mutants made in the *msh2*-*Δ* background that had amplified ApaL repeats might also be related to mismatch repair. Alternatively, we cannot rule out the possibility that the increased incorporation of ApaL repeats occurring in the precursor during the additional cell divisions (equivalent to 2–3 streaks) needed to disrupt the *MSH2* gene altered the result.

We speculated that *stn1-M1 ter1-Δ* mutants made in a *msh2*-*Δ* background had sufficiently high levels of telomeric recombination to rapidly break down any repeating structure that might have been formed initially. To test this idea, we attempted to knock out the *MSH2* gene in the A1 clone of *stn1-M1 ter1-Δ* mutant that had highly favored small telomeric fragments in both *Apa*LI and *Rsa*I digests (Figure 1D). Among 72 clones transformed with the knockout cassette, only one had the *MSH2* gene successfully disrupted. As homologous gene disruption rate, even with the fragment used in this experiment, is generally 10–40% in *STN1* cells, it is conceivable that very high levels of telomeric recombination might interfere with homologous recombination elsewhere in the genome of *stn1-M1* cells. The one *stn1-M1 msh2*-*Δ* clone we did recover was serially passaged on YPD plates for three streaks and its telomeres were examined after each streak by Southern blot (Figure 2B). The same procedure was carried out with a control transformant that still had the intact *MSH2* gene. Upon disruption of *MSH2*, the simple banding patterns of the A1 clone, particularly the larger fragment in *Apa*LI digests, become distinctly heterogeneous as soon as the samples could be analyzed (Figure 2B). This heterogeneous pattern is similar to those from *stn1-M1 ter1-Δ* mutants generated directly in a *msh2*-*Δ* background shown in Figure 2A. In contrast, the control transformant having the intact *MSH2* exhibited a banding pattern that remained distinctly more stable. This result demonstrates that the elevated telomeric recombination in a *msh2*-*Δ* background is sufficient enough to rapidly break down a repeating structure that might have been formed initially in telomeres of a newly generated *stn1-M1 ter1-Δ* mutant. A diagram summarizing our experiments with *stn1-M1 ter1-Δ* mutants generated from ApaL precursors is shown in Figure 2C.

### The sequence from a single telomere can be spread to all other telomeres during the establishment of the *stn1-M1 ter1-Δ* state

**Figure 3:**
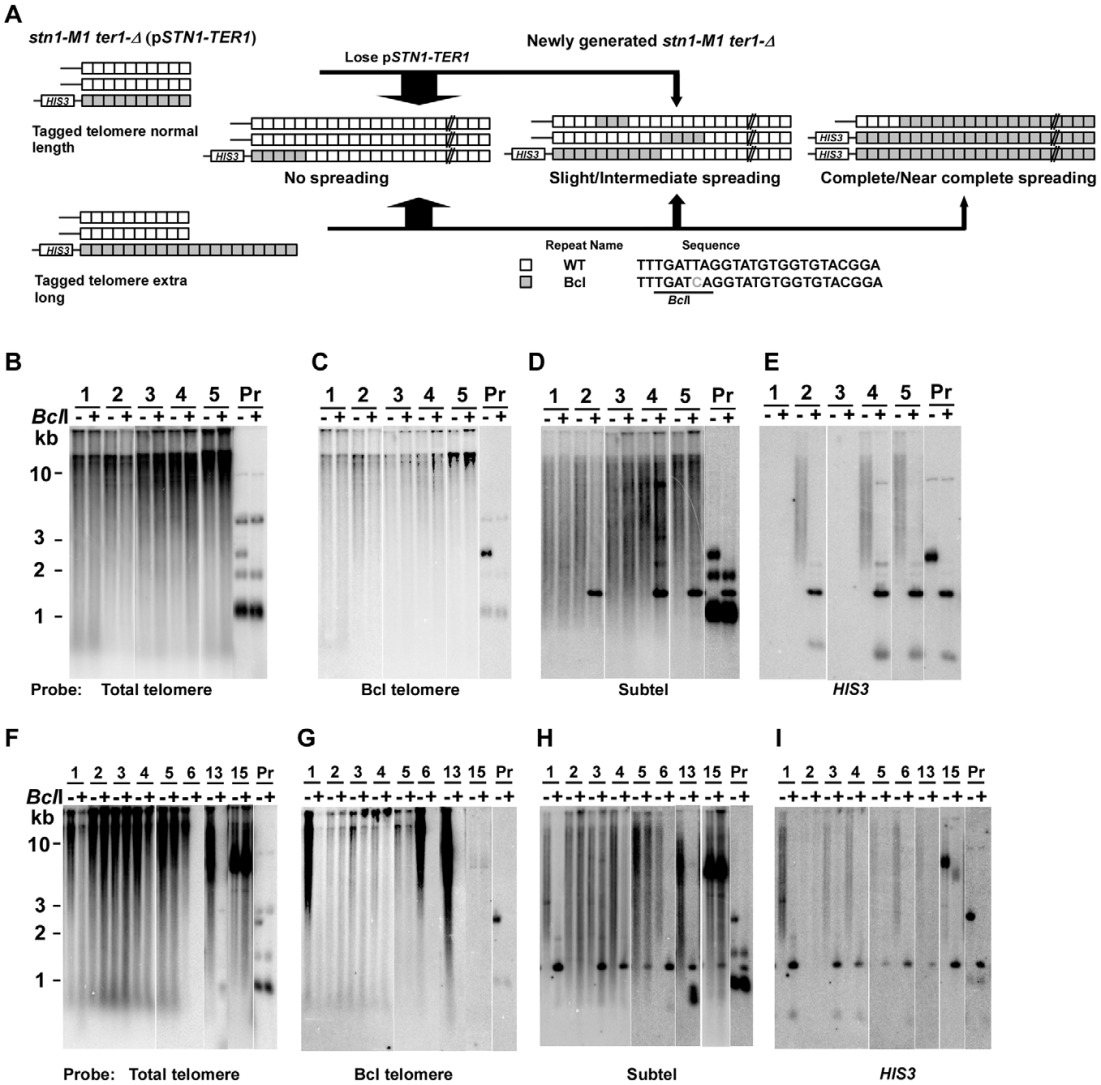
Spreading of a telomeric sequence from a single telomere source during RTE in *stn1-M1 ter1-Δ* cells. (A) Experimental outline showing generation of *stn1-M1 ter1-Δ* mutants from *STN1 TER1* precursors containing a single telomere of either normal length or ∼2× longer than normal length that are composed of Bcl repeats. The sequence of the Bcl repeat is indicated. (B) Southern blot, hybridized with a telomere probe, of *Eco*RI (indicated by “−”) and *Eco*RI+*Bcl*I (indicated by “+”) digested DNA from 5 newly generated *stn1-M1 ter1-Δ* clones from normal length Bcl precursors as well as from one normal length Bcl precursor *STN1 TER1* control. (C–E) Same filter as in panel B after stripping and rehybridization with a Bcl telomere probe, subtelomeric probe and *HIS3* probe, respectively. (F) Southern blot, hybridized with a telomere probe, of *Eco*RI (indicated by “−”) and *Eco*RI+*Bcl*I (indicated by “+”) digested DNA from 8 newly generated *stn1-M1 ter1-Δ* clones from long Bcl precursors as well as from one long Bcl precursor *STN1 TER1* control. (G–I) Same filter as in panel F after stripping and rehybridization with a Bcl telomere probe, subtelomeric probe and *HIS3* probe, respectively.

RTE in *ter1-Δ* mutants of *K. lactis* regularly involves the spreading of sequence from a single telomere to all other telomeres to generate modest elongated telomeres [37]. To test whether the *stn1-M1* mutant can also use this mechanism to generate highly elongated telomeres, we constructed telomeric DNA fragments consisting of the subtelomeric sequence, a *HIS3* marker and Bcl telomeric repeats (Figure 3A). The subtelomeric sequence is shared by 11 of 12 *K. lactis* telomeres allowing the transformed fragments to replace a native telomere through homologous recombination. The Bcl repeats each contain a phenotypically silent base change that generates a *Bcl*I site [51]–[53]. DNA fragments containing either ∼11 or ∼40 Bcl repeats were transformed into *stn1-M1 ter1-Δ* cells containing p*STN1*-*TER1* integrated at the *stn1-M1* locus (Figure 3A). While the shorter telomeric fragment generated a new telomere of normal length (Figure 3B), the long telomeric fragment generated a telomere substantially longer than those of wild type cells (Figure 3F). The resulting transformants are hereafter referred to as “normal length Bcl precursors” and “long Bcl precursors”, respectively.

**Figure 4:**
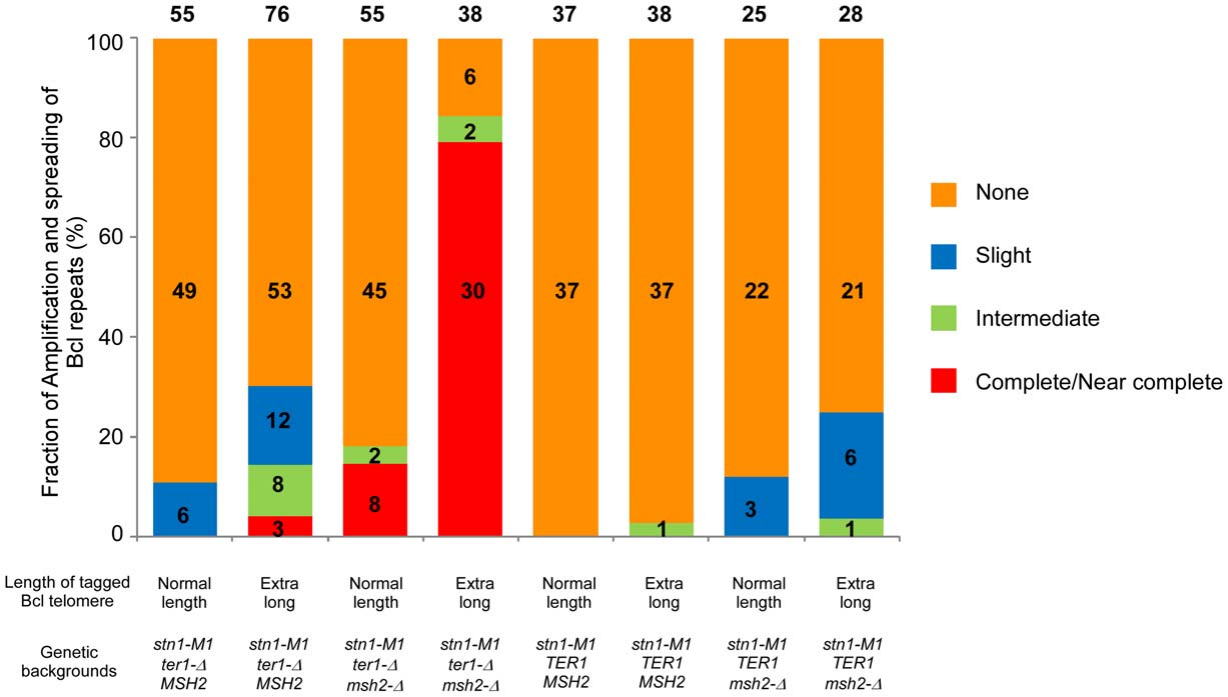
Frequency of spreading of sequence from a single tagged telomere to other telomeres in *K. lactis stn1-M1* mutants with different genetic backgrounds. Columns 1–8 show histograms of the frequencies of four different types of outcomes (none, slight, intermediate and complete/near complete) with regard to the extent and spreading of ApaL repeats in newly generated *stn1-M1* mutants. Numbers above each column indicate the total number of clones examined. Numbers within histograms indicate the number of clones of each type when those numbers were above zero. Definitions of the outcome types are as follows. None: defined by each of the following being true: 1) total *Eco*RI-digested telomeric signal is reduced no more than 1.5 fold by additional *Bcl*I digestion; 2) Bcl specific telomeric signal is less than 2.5 fold more than that of “precursor”; 3) *Eco*RI-digested subtelomeric signal larger than 2 kb is reduced no more than 1.5 fold by *Bcl*I digestion. Slight: defined by each of the following being true: 1) total *Eco*RI-digested telomeric signal is reduced no more than 3 fold by additional *Bcl*I digestion; 2) Bcl specific telomeric signal is less than 5 fold more than that of “precursor”; 3) *Eco*RI-digested subtelomeric signal larger than 2 kb is reduced more than 1.5 fold by *Bcl*I digestion. Intermediate: defined by each of the following being true: 1) total *Eco*RI-digested telomeric signal is reduced no more than 3 fold by additional *Bcl*I digestion; 2) Bcl specific telomeric signal is 5 fold or more higher than that of “precursor”; 3) *Eco*RI-digested subtelomeric signal larger than 2 kb is reduced more than 1.5 fold by *Bcl*I digestion. Complete/Near Complete: defined by each of the following being true: 1) total *Eco*RI- digested telomeric signal is reduced at least 3 fold by additional *Bcl*I digestion; 2) Bcl specific telomeric signal is 8 fold or more than that of “precursors”; 3) *Eco*RI-digested subtelomeric signal larger than 2 kb is reduced more than 1.5 fold by *Bcl*I digestion.

Next, we generated 55 clones of *stn1-M1 ter1-Δ* mutants from four normal length Bcl precursors by plating the precursors on 5-FOA-containing medium that selected for the loss of the p*STN1*-*TER1* plasmid. Telomeric signals in 49 of these clones appear essentially identical in both *Eco*RI and *Eco*RI+*Bcl*I digests (Clones 1, 3, 4 and 5 in Figure 3B and data not shown) which indicated that telomeres in these clones were composed mostly or entirely of WT repeats with very few or no Bcl repeats. Other data were also consistent with this view. The weak signal observed in these clones with a probe specific to Bcl telomeric repeats was not sensitive to *Bcl*I digestion, suggesting that it was due to background hybridization to the heavily amplified wild type repeats (Figure 3C). Additionally, subtelomeric signals in these clones were largely resistant to *Bcl*I digestion, except for ∼1.5 kb fragments generated in some clones (Clones 2, 4 and 5 in Figure 3D–3E) that also hybridized to *HIS3* probe, consistent with them being derived from the original integrated telomere. We classified these 49 clones as having no amplification or spreading of Bcl repeats (Figure 4, column 1). Our results suggest that Bcl repeats were actively avoided as a source of sequence to be amplified during establishing the long telomeres in the *stn1-M1 ter1-Δ* mutant.

The remaining 6 of 55 clones, including clone 2 in Figure 3B, showed amplification of Bcl repeats estimated to be 3–5 times that present in the single telomere of the precursor strain (Figure 4 and data not shown). At least two of these clones showed extra copies of the *HIS3* gene (data not shown), suggesting that the modest amplification of Bcl repeats occurred primarily by subtelomeric break-induced replication (BIR) events that produced extra copies of the tagged telomere but without the Bcl repeats otherwise becoming amplified.

Of the 55 total clones, 41 (including 38 of 49 clones with no amplification and spreading of Bcl repeats) showed no *HIS3* signal (Figure 3E and data not shown). This indicates that these mutants lost the *HIS3*-tagged telomere and probably all the Bcl repeats originally attached to that telomere. Such frequent loss of the subtelomeric *HIS3* was not entirely surprising as that *stn1-M1* cells showed very high rates of loss of a *URA3* gene inserted at the same subtelomeric location [42]. These deletions are likely BIR events that replace one telomere with sequence from another telomere. The same mechanism is likely responsible for occasions in some mutants where the *HIS3*-tagged telomere became duplicated.

Analysis of 76 newly generated clones of *stn1-M1 ter1-Δ* mutants from long Bcl precursors showed somewhat different results (Figure 4, column 2). While 53 clones (70%) showed no amplification or spreading of Bcl repeats, the other 23 clones (30% of the total) did show some degree of amplification and spreading of Bcl repeats. Most notably, telomeric signals in three of these clones, including clones 6 and 13 in Figure 3F, can be completely or nearly completely cleaved away by *Bcl*I digestion, suggesting that telomeres in these clones were composed mostly or entirely of Bcl repeats (Figure 4). Consistent with this interpretation, the Bcl repeat-specific signals of these three clones were intense in *Eco*RI digests but were eliminated by *Bcl*I digestion (Figure 3G). Furthermore, the long smeared subtelomeric signal of these clones in *Eco*RI digests was largely or entirely cleaved by *Bcl*I into fragments that were generally <2 kb (Figure 3H). Interestingly, these three clones showed no amplification of the subtelomeric *HIS3* gene (Figure 3I and data not shown), suggesting that the Bcl repeats were amplified by inter-telomeric recombination rather than by subtelomeric duplication.

As indicated in Figure 4, 8 of the 76 clones derived from the long Bcl precursor (including clone 1 in Figure 3F–3I) were classified as having intermediate amplification and spreading of Bcl repeats based on: 1) total telomeric signal that was partially cleaved by *Bcl*I (Figure 3F); 2) Bcl-specific telomeric signal that was at least 5 times that of the precursor (Figure 3G and data not shown); and 3) subtelomeric signal that was substantially cleaved into one or more short fragments by *Bcl*I digestion (Figure 3H). These results suggest that both Bcl and WT repeats were amplified and copied onto multiple telomeres in these mutants. This group of clones had no obvious telomeric fragments of 50–500 bp in *Eco*RI+*Bcl*I digests (Figure 3F and data not shown), suggesting that the amplified Bcl and WT repeats were not generally interspersed closely together. Indeed, the signal from WT repeats that remained after *Bcl*I digestion was generally very long, suggesting that WT repeats in these clones were often in long continuous arrays (Figure 3F and data not shown). Twelve other clones (including clone 4 in Figure 3F–3I) were classified as having slight amplification and spreading of Bcl repeats based on lesser degrees of both amplification of Bcl repeats and subtelomeric signal that was cleaved by *Bcl*I (Figure 4, column 2).

One clone of the *stn1-M1 ter1-Δ* mutant, clone 15 in Figure 3F–3I, showed a unique outcome. It displayed a ∼9–10 kb band that hybridized intensely with both telomeric and subtelomeric probes but was resistant to *Bcl*I digestion (Figure 3F and 3H). These results are consistent with the possibility that this clone contained tandem arrays composed of both WT telomeric repeats and subtelomeric sequences, but not Bcl repeats. *HIS3* was detected in the clone but apparently was not within the amplified fragment (Figure 3I). Conceivably, the putative tandem arrays originated from a native telomere rather than the Bcl telomere. This clone is reminiscent of type I post-senescent survivors in *Sacchromyces cerevisiae*, which are characterized by amplified subtelomeric Y′ elements and short tracts of telomeric repeats [31], [54]. Although type I-like survivors with alternating telomere and non-telomeric sequences can occur in *K. lactis* cells that are transformed with a circle containing telomeric repeats and the *URA3* gene [35], [48], to our knowledge, this is the first report of RTE amplifying subtelomeric sequences in *K. lactis*.

23 of 76 *stn1-M1 ter1-Δ* mutants (30%) derived from long Bcl precursors had lost *HIS3* signal (Figure 3I and data not shown). All 23 of these clones showed no amplification and spreading of Bcl repeats. This loss rate of *HIS3* was significantly less (p<0.001, in Fisher exact test) than that seen in mutants derived from normal Bcl precursors where 41 of 55 clones (75%) showed no *HIS3* signal. This result suggests that a long telomere stabilizes the adjacent subtelomeric sequences from being lost during the establishment of an *stn1-M1 ter1-Δ* mutant.

### The absence of mismatch repair facilitates the spreading of sequence from a single tagged telomere to other telomeres

**Figure 5:**
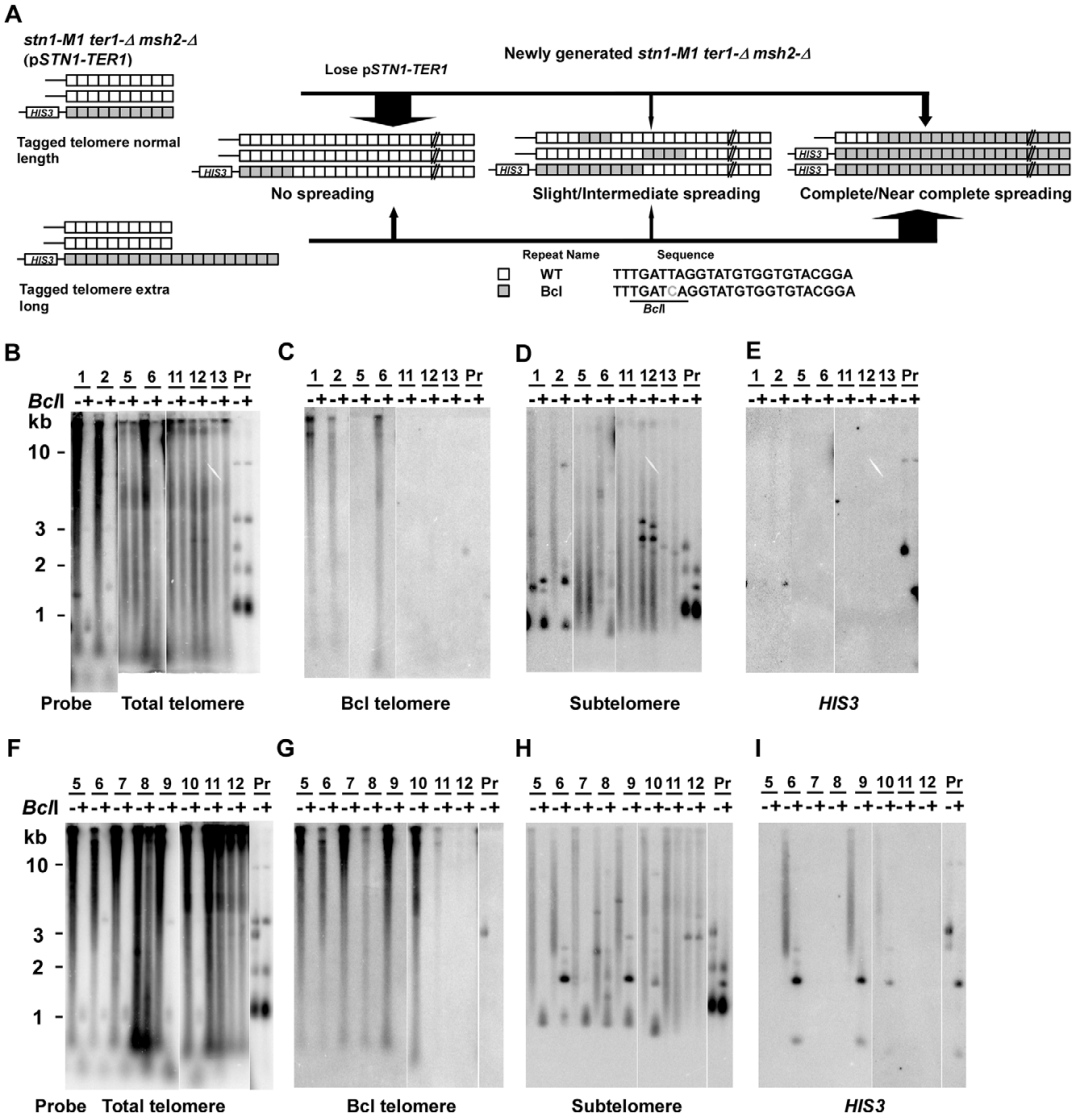
Spreading of a telomeric sequence from a single telomere source during RTE in *stn1-M1 ter1-Δ msh2-Δ* cells. (A) Experimental outline showing generation of *stn1-M1 ter1-Δ msh2-Δ* mutants from *STN1 TER1 msh2-Δ* precursors containing a single telomere of either normal length or ∼2× longer than normal length that are composed of Bcl repeats. The sequences of a WT repeat and a Bcl repeat are indicated. (B) Southern blot, hybridized with a telomere probe, of *Eco*RI (indicated by “−”) and *Eco*RI+*Bcl*I (indicated by “+”) digested DNA from 7 newly generated *stn1-M1 ter1-Δ msh2-Δ* clones from normal length Bcl precursors in a *msh2-Δ* background as well as one normal length Bcl *STN1 TER1 msh2-Δ* precursor control. (C–E) Same filter as in panel B after stripping and rehybridization with a Bcl telomere probe, subtelomeric probe and *HIS3* probe, respectively. (F) Southern blot, hybridized with a telomere probe, of *Eco*RI (indicated by “−”) and *Eco*RI+*Bcl*I (indicated by “+”) digested DNA from 8 newly generated *stn1-M1 ter1-Δ msh2-Δ* clones from long Bcl precursors in a *msh2-Δ* background as well as one long Bcl *STN1 TER1msh2-Δ* control. (G–I) Same filter as in panel F after stripping and rehybridization with a Bcl telomere probe, subtelomeric probe and *HIS3* probe, respectively.

To test whether the low frequency of spreading Bcl repeats to other telomeres in *stn1-M1 ter1-Δ* mutants was affected by mismatch repair, we constructed normal length Bcl precursor and long Bcl precursor strains containing p*STN1*-*TER1* and a *msh2*-*Δ* mutation (Figure 5A). After screening for loss of p*STN1*-*TER1*, we identified 55 *stn1-M1 ter1-Δ msh2*-*Δ* mutants from three normal length Bcl precursors and 38 mutants from two long Bcl precursors. The results showed that the *msh2*-*Δ* background permitted a substantially higher frequency of amplification and spreading of Bcl repeats than a *MSH2* background did. Eight of 55 *stn1-M1 ter1-Δ msh2*-*Δ* mutants (15%) derived from normal length Bcl precursors (including clones 1–2 in Figure 5B–5E) showed complete or near complete spreading of Bcl repeats (Figure 4, column 3). Two mutants (4%), including clone 6 in Figure 5B–5E, showed intermediate amplification and spreading of Bcl repeats and the remaining mutants (45 of 55; 81%), including clones 5 and 11–13 in Figure 5B–5E, showed no amplification (Figure 4, column 3).

Remarkably, 30 of 38 mutants (78%) derived from long Bcl precursors (including clones 5–7, 9, and 10 in Figure 5F–5I) showed complete or near complete spreading of Bcl repeats (Figure 4, column 4). At least 25 of these clones appeared to contain a small percentage of WT repeats interspersed among the Bcl repeats, as indicated by telomeric signal of <500 bp present in *Eco*RI+*Bcl*I digestion (e.g., clones 5–7, 9, and 10 in Figure 5F). However, as these telomeric signals were not intense, it is likely that the WT repeats were not interspersed throughout the telomeres. Only two clones (6%), including clone 8 and 11 in Figure 5F–5I, showed intermediate amplification and spreading of Bcl repeats (Figure 4, column 4). As the disruption of mismatch repair presumably permits Bcl repeats to recombine with WT repeats in an unperturbed fashion, we conclude that the long telomeres formed during the establishment of an *stn1-M1 ter1-Δ* mutant are regularly derived primarily from a single telomere source.

45 of 55 (82%) *stn1-M1 ter1-Δ msh2*-*Δ* mutants derived from normal Bcl precursors (Figure 5E and data not shown) and 21 of 38 (55%) mutants derived from long Bcl precursors (Figure 5I and data not shown) had lost the *HIS3* genes. This difference was significant (p = 0.0005 in Fisher exact test). Strikingly, many clones that had complete or nearly complete spreading of Bcl repeats (4 of 8 mutants from the normal length Bcl precursor and 14 of 30 from the long Bcl precursor) had lost the *HIS3* genes (clone 5 and 7 in Figure 5I and data not shown). On the other hand, 4 of 18 clones that had complete or near complete spreading of Bcl repeats contained estimated 5–10 copies of *HIS3* genes (data not shown). These results showed that the spreading of Bcl repeats to all other telomeres could occur either with or without concurrent spreading of the subtelomeric *HIS3* to other telomeres.

### The presence of telomerase inhibits the spreading of sequence from a single tagged-telomere to other telomeres

**Figure 6:**
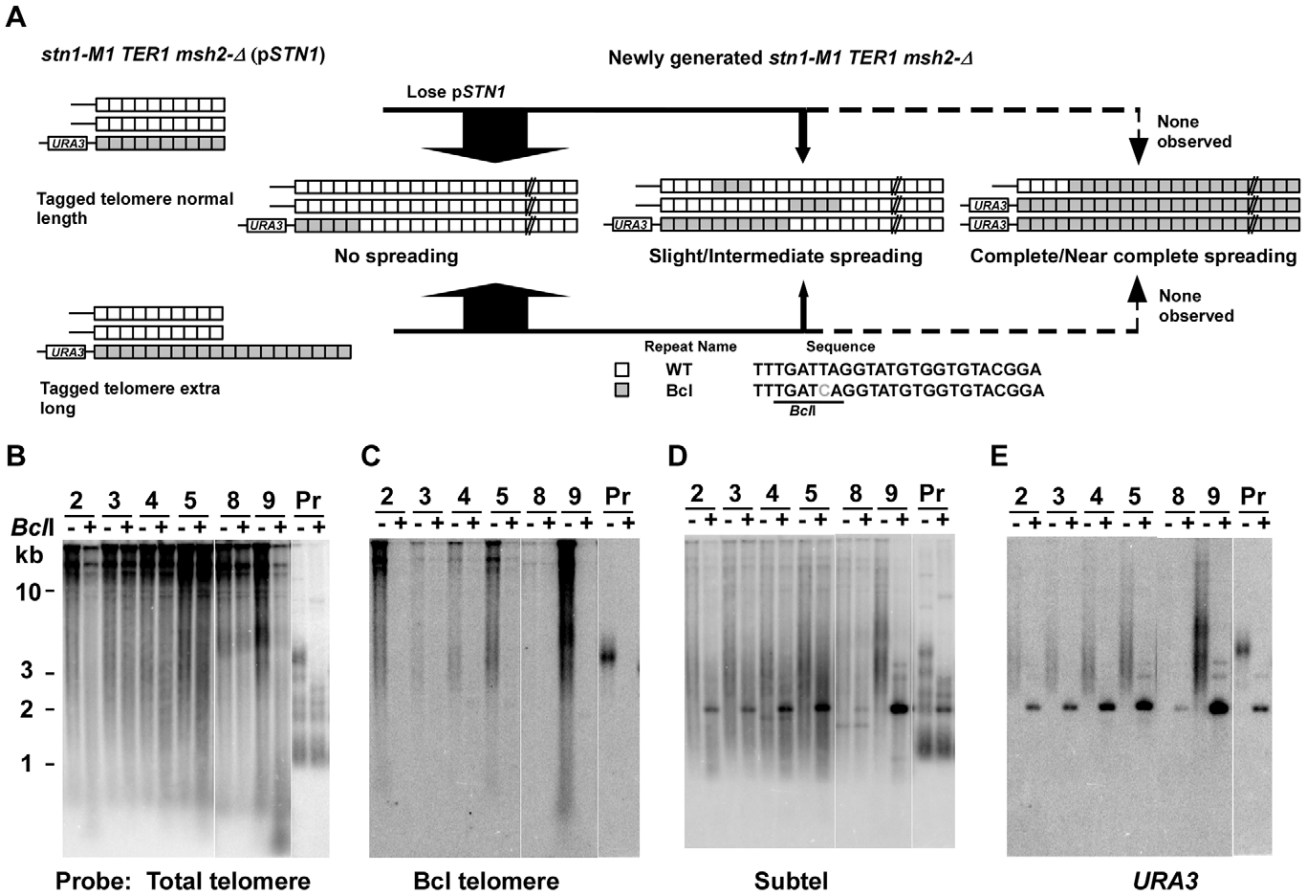
Spreading of a telomeric sequence form a single telomere source during RTE in *stn1-M1 TER1 msh2-Δ* cells. (A) Experimental outline showing generation of *stn1-M1 TER1 msh2-Δ* mutants from *STN1 TER1 msh2-Δ* precursors containing a single telomere composed of Bcl repeats that is either normal length or ∼2× longer than normal length. The sequences of a WT repeat and a Bcl repeat are indicated. (B) Southern blot, hybridized with a telomere probe, of *Eco*RI (indicated by “−”) and *Eco*RI+*Bcl*I (indicated by “+”) digested DNA from six newly generated *stn1-M1 TER1 msh2-Δ* clones from long Bcl precursors in a *TER1 msh2-Δ* background as well as one normal length Bcl *STN1 TER1 msh2-Δ* precursor control. (C–E) Same filter as in panel B after stripping and rehybridization with a Bcl telomere probe, subtelomeric probe and *URA3* probe, respectively.

To test the effect of telomerase on amplification and spreading of Bcl repeats, we constructed normal length Bcl precursors and long Bcl precursors in both *stn1-M1 TER1 MSH2* and *stn1-M1 TER1 msh2*-*Δ* backgrounds that were complemented by plasmid p*STN1*. These precursors were similar to those used above except that the subtelomeric marker gene was *URA3* and the complementing plasmid carried *STN1* and *HIS3* (Figure 6A). We generated *stn1-M1* mutants from all four precursor types by screening for loss of p*STN1* after streaking onto YPD plates and identifying clones with the rough colony phenotype characteristic of *stn1-M1* mutants. Results from this showed that telomerase significantly inhibited the amplification and spread of Bcl repeats (Figure 6; Figure S3; and Figure 4, columns 5–6). None of 37 *stn1-M1 TER1 MSH2* clones derived from normal-length precursors showed amplification and spreading of Bcl repeats (Figure S3A–S3D and Figure 4, column 5), and only 1 of 38 *stn1-M1 TER1 MSH2* clones from the long precursors showed detectable amplification and spreading (clone 9 in Figure S3E–S3H and Figure 4, column 6). The one mutant that did show amplification (an intermediate level) was estimated to contain three copies of *URA3* (data not shown). This may suggest that amplification and spreading of Bcl repeats in this clone occurred primarily through subtelomeric BIR events that produced extra copies of the tagged telomere rather than by telomere-telomere recombination.

Similar outcomes were seen with *stn1-M1 TER1 msh2*-*Δ* mutants. There, the frequency of amplification and spreading of Bcl repeats was markedly lower than that seen in *stn1-M1 ter1-Δ msh2*-*Δ* mutants (Figure 4, columns 7–8). None of 28 *stn1-M1 TER1 msh2-Δ* mutants obtained from the long precursors showed complete amplification and spreading of Bcl repeats, and only 7 of 28 mutants showed intermediate or slight amplification. Clone 9 in Figure 6B–6E is the sole example of intermediate amplification. Among *stn1-M1 TER1 msh2*-*Δ* mutants derived from a normal length Bcl precursor, only 3 of 25 mutants, including clones 6 and 7 in Figure S3I–S3L, showed slight Bcl amplification (Figure 4, column 7).

The subtelomeric *URA3* marker was comparatively stable in the *stn1-M1 TER1* mutants. Only 7 of 67 *stn1-M1 TER1 msh2*-*Δ* mutants and no *stn1-M1 TER1 MSH2* mutants (0 of 39) had lost *URA3* (Figure S3D, S3H, S3L; Figure 6E; and data not shown). These results suggest that an active telomerase in *stn1-M1* mutants helps to stabilize the adjacent subtelomeric sequences against loss or amplification.

## Discussion

Accumulated previous evidence suggests that a roll-and-spread mechanism is involved in generating the moderately elongated telomeres formed in *K. lactis ter1-Δ* post-senescence survivors (reviewed in [27]). Our studies here suggest that this mechanism, involving amplification of sequence from a single t-circle is also involved in establishing the more ALT-like highly elongated telomeres in the *K. lactis stn1-M1* mutant. Some support for the importance of t-circles in *stn1-M1* cells already existed. Previous data had shown that t-circles are abundant in *stn1-M1* cells [38]. Additionally, a recent study showed that introducing t-circles into *stn1-M1* cells leads to tandem arrays of the circle's sequence becoming incorporated at multiple telomeres [48].

The use of mutationally tagged repeats was critical to earlier studies on how RTE occurs in a *ter1-Δ* mutant [35]–[37]. However, the type II RTE in this mutant is episodic, occurring when telomeres drop below a critical length and essentially shutting off once telomeres are moderately lengthened [24]. The transient stability of the lengthened telomeres allowed Southern blots that examined the structure of telomeric DNA from populations of cells to be very informative. We anticipated that the telomerase-resistant type IIR RTE of the *stn1-M1* mutant, with its apparently continuous high rate of telomeric recombination, would be more problematic to study by this method as any long telomere initially generated would be expected to be unstable and would become altered by further recombination events in many if not most cells of any culture large enough to be studied. The first tagging approach used in this study involved generating *stn1-M1 MSH2* mutants from precursors with ApaL repeats at all telomeric termini. Among the mutant clones that had both amplified WT and ApaL repeats, roughly half had telomeric blocks of both types of repeats that had single favored sizes. Although other interpretations cannot be ruled out, this result is consistent with a roll-and-spread mechanism that derived all amplified telomeres from the sequence of a single small t-circle if the uniformly repeating patterns predicted from rolling circle copying of a t-circle containing both WT and ApaL repeats had been extensively disrupted by later recombination events.

Other results clearly demonstrated that ongoing recombination in *stn1-M1* mutants can disrupt existing signs of repeating patterns. This was most strikingly seen when a *stn1-M1 ter1-Δ MSH2* mutant with a very non-random WT/ApaL repeat pattern had its *MSH2* gene disrupted. As soon as this *msh2-Δ* derivative could be examined, its telomeric repeats had acquired a much more randomized pattern similar to those seen in *stn1-M1 ter1-Δ* mutants established directly in a *msh2-Δ* background. As discussed further below, loss of mismatch repair is expected to elevate recombination rates between WT and ApaL repeats to levels that would occur if no mismatches were present. Thus, even the complete absence of preferred sizes of blocks of WT and ApaL repeats seen in *stn1-M1 ter1-Δ msh2-Δ* clones is not inconsistent with a single t-circle having been the original source of the amplified telomeres.

A result of particular importance in our study was that Bcl repeats present at a single telomere in a precursor cell were sometimes the source of virtually all of the amplified telomeric DNA sequences in newly generated *stn1-M1* mutants. This effect was most prominent in a strain lacking both telomerase and *MSH2*. This mutant combination is probably the most relevant of those examined that used Bcl repeats to study telomeric recombination. This is because the absence of telomerase assures that all telomeric lengthening is due to recombination while the absence of mismatch repair presumably permits recombination between wild type and Bcl telomeres to occur at the same frequency as recombination between two wild type telomeres in otherwise equivalent circumstances. In the *ter1-Δ msh2-Δ* strain, the percentage of *stn1-M1* mutants that displayed complete or near complete amplification and spreading of Bcl repeats was 14% in mutants derived from the normal length Bcl precursors and 78% in mutants derived from the long Bcl precursors (Figure 4). These results are very similar to those seen in an earlier study with *K. lactis ter1-Δ* mutants, where total spreading of Bcl repeats to all telomeres from a single normal length Bcl telomere and a single long Bcl telomere was measured at 10% and 94%, respectively [37]. As was proposed with *ter1-Δ* mutants, we suggest that a roll-and-spread mechanism, where rolling circle amplification of a single t-circle followed by spreading of that sequence to all telomeres by BIR events, can account for these observations. When all twelve telomeres are the same length and each has the same chance to be amplified and spread, the predicted frequency of clones where Bcl repeats have been amplified and spread would be roughly one twelfth. The much greater frequency of spreading of Bcl repeats that is observed from mutants derived from the long Bcl precursors indicates that a longer telomere is much better able than a shorter telomere at promoting the spread of its sequence to other telomeres. The roll-and-spread model predicts that this could occur because the long Bcl telomere is used directly as a template for lengthening other telomeres and/or is better at forming t-circles. Although t-circles are common in established *stn1-M1* cells, the fact that all amplified telomeric sequences can be derived from a single telomere may suggest that t-circle formation is limiting during the initial establishment of the mutant state. Additionally, the copying of sequence from a lengthened telomere onto other telomeric ends might be facilitated by the physical clustering of uncapped telomeres. In favor of this, it has been reported that multiple double-strand DNA breaks (DSBs) are often recruited to a single Rad52-containing focus for DNA repair in *S. cerevisiae* [55].

The amplification and spreading of Bcl repeats in newly generated *stn1-M1* mutants was strongly inhibited by the mismatch repair system. This is not surprising as mismatch repair can inhibit recombination involving sequences with imperfect homology [50]. For example, in *S. cerevisiae*, 1% and 6% sequence divergence can reduce mitotic recombination ∼20 and ∼140 fold, respectively [56]. Therefore, the 4% sequence divergence between Bcl repeats and WT repeats in our study may significantly reduce the recombination between the telomeres containing them. Mismatch repair has been suggested to inhibit recombination by rejecting or processing the heteroduplex formed by strand invasion occurring with homeologous sequences [50]. Loss of *MSH2* was previously shown to facilitate the recombination that generates post-senescence survivors in telomerase deletion mutants of *S. cerevisiae* and *K. lactis* [57]. This was attributed to increased recombination between the homeologous telomeric repeats in *S. cerevisiae* and between the homeologous subtelomeric sequences in *K. lactis*. Deficiency in mismatch repair can also facilitate ALT-like telomerase-independent telomere elongation in human colon cancer cell lines and in gastric carcinomas [20], [58]. Conceivably, this effect stems from the degenerate telomeric repeats that are common in the basal regions of human telomeres [59]–[60].

In *stn1-M1* mutants formed in a *ter1-Δ MSH2* background, there was not only a substantial reduction in the frequency of clones exhibiting total spreading of Bcl repeats but also, when spreading was observed, it was far more likely to be partial, accompanied by substantial amplification of WT repeats as well. We suggest that in these clones, formation of a t-circle from the Bcl telomere will occur efficiently (as no mismatches would be present during intratelomeric recombination) but copying sequence from a t-circle composed purely of Bcl repeats onto any of the 11 wild type telomeres would be impeded. When copying of a Bcl repeat t-circle did occur, the impeded ability of the Bcl repeats to recombine with the resident wild type telomeres could allow time for wild type t-circles to form and have their sequences amplified.

Interestingly, mismatch repair was apparently not an appreciable barrier to recombination between Bcl and WT repeats in *STN1* during the formation of post-senescence survivors in *ter1-Δ* mutants [37]. One possibility to account for this is that the extremely short telomeres in senescing cells of these mutants recombine in a different way than the somewhat longer telomeres in newly forming *stn1-M1* mutants. This is supported by the fact that the type II RTE in *S. cerevisiae* depends on a pathway involving Rad50 when occurring at very short telomeres in a senescing telomerase deletion mutant but depends on the more standard Rad51-dependent pathway when occurring at longer telomeres uncapped by defects in telomere capping proteins [30], [40]. Also, Rad51-dependent recombination is inhibited by mismatch repair 13-fold more than Rad50-dependent recombination [61]. The formation of post-senescence survivors in *K. lactis STN1 ter1-Δ* mutants has recently been shown to require both the Rad50- and the Rad51- pathways (Basenko and McEachern, unpublished data).

Telomerase is active in *stn1-M1* cells, but its presence does not grossly affect the phenotype of the mutant [42]. This indicates that the recombination by itself is capable of generating and maintaining the very long and unstable telomeres of *stn1-M1* cells and that the recombinational processes of type IIR RTE in *stn1-M1* cells are not suppressed by the presence of telomerase. Our results here demonstrate that the presence of telomerase at the establishment of the *stn1-M1* state can largely inhibit the amplification and spreading of Bcl repeats. While we cannot rule out the possibility that telomerase fundamentally alters the mechanism by which telomeres are maintained in *stn1-M1* cells, we believe this is unlikely. Rather, we suggest that sequence addition by telomerase masks the effects of recombination. In particular, we suggest that the *stn1-M1* mutation, like certain telomeric repeat mutations shown to produce type IIR RTE, is disrupted not only in telomeres' ability to block recombination but also in their ability to negatively regulate sequence addition by telomerase [37], [41]. Consistent with this possibility is our observation that telomerase inhibits the spreading of Bcl repeats not only from a normal length telomere, but also from long Bcl telomere in *stn1-M1* cells. The latter is resistant to telomerase addition in wild type *K. lactis* cells [37]. With multiple telomerase-synthesized WT telomeric repeats added onto the ends of both long and normal length Bcl telomeres, t-circles formed from terminal deletions of these telomeres would likely often be composed only of WT repeats. This would of course interfere with the ability of the Bcl repeats to amplify and spread to other telomeres. The relative contribution of recombination and telomerase to new telomeric repeat synthesis in *stn1-M1 TER1* cells is not fully known. In experiments we performed where *TER1-20C(ApaL)* was present during the establishment of newly generated *stn1-M1* mutants, we found that ApaL repeats were present but only as a minority of the telomeric repeats in each of multiple clones examined (data not shown). This argues that recombination is the predominant mechanism for telomeric repeat amplification in *stn1-M1* cells. It is reasonable to believe, however, that the contribution of telomerase might be greatest at the earliest stages of formation of the *stn1-M1* state, before recombination has abundant long telomeric sequences available for it to copy.

One unexpected result from our work was that precursor cells with ApaL repeats present at the termini of all telomeric ends produced *stn1-M1* mutants where the ApaL repeats were generally completely absent. This occurred in both *msh2-Δ* and *MSH2* backgrounds and therefore does not appear to require mismatch repair. As the ApaL telomeric base change does not appear to alter telomere function [49], this effect is also not likely to be due to selection against amplification of ApaL repeats. The simplest explanation for this outcome would be that a significant fraction of telomeric termini, at least a third of the ∼350–600 bp telomeres by our estimates, is routinely deleted prior to the initiation of recombination events that elongate telomeres when the *stn1-M1* mutant state is established. Telomere shortening also precedes RTE in yeast telomerase deletion mutants. In that case, however, the shortening occurs very gradually over many tens of cell divisions from replicative sequence loss [54], [62]–[63]. Such gradual telomere shortening cannot explain the terminal sequences loss we see in *stn1-M1* cells. Most newly generated spores of the *stn1-M1* mutant die within a few cell divisions [42]. This indicates that they experience a severe growth problem immediately after their generation and suggests that the terminal telomeric loss occurs very rapidly. Indeed, the rapid telomeric deletion might help account for the poor viability of *stn1-M1* spores.

A number of mechanisms have been proposed for generating telomeric deletions (for a review see [64]). One well studied mechanism is telomere rapid deletion (TRD) which is thought to be an intratelomeric recombination event that occurs after a telomeric 3′ overhang strand invades into the double-stranded region of the same telomere (for a review see [65]). TRD can lead to telomere deletion in wild type yeast cells that contain artificially elongated telomeres [66]–[68]. Processes similar to TRD, requiring the recombination protein XRCC3, can lead to shortening of both normal and dysfunctional mammalian telomeres [18], [69]. TRD has been proposed to be a mechanism that can generate t-circles [34], [68].

An obvious question that arises from our data is why telomeric truncations would predominate at the earliest stage of the formation of a mutant that ultimately generates and maintains highly elongated telomeres. At least two possibilities exist. One is that the physiological conditions at the earliest stage of *stn1-M1* mutants, when telomere deletions occur, are different from those at later stages, when telomere elongation predominates. Conceivably, later stages might be influenced by the presence of chronic DNA damage and react differently to dysfunctional telomeres compared to cells at the earliest stage. This idea is supported by the finding that *S. cerevisiae* telomerase deletion mutants showed expression changes in hundreds of genes during the senescence caused by shorting telomeres [70]. Another possibility is that telomeric deletions are always more frequent than recombination events that lengthen telomeres in *stn1-M1* cells. In this scenario, net lengthening might only predominate over shortening once long telomeres or t-circles are present and abundant enough to serve as efficient templates for elongation events that can generate long extensions. Some observations support this possibility as well. In wild type *K. lactis* cells, deletions from TRD are approximately an order of magnitude more frequent than telomere elongation by recombination [67]. Also, telomeric deletions are very frequent in *stn1-M1* cells and in other mutants undergoing type IIR RTE [41], [48].

**Figure 7:**
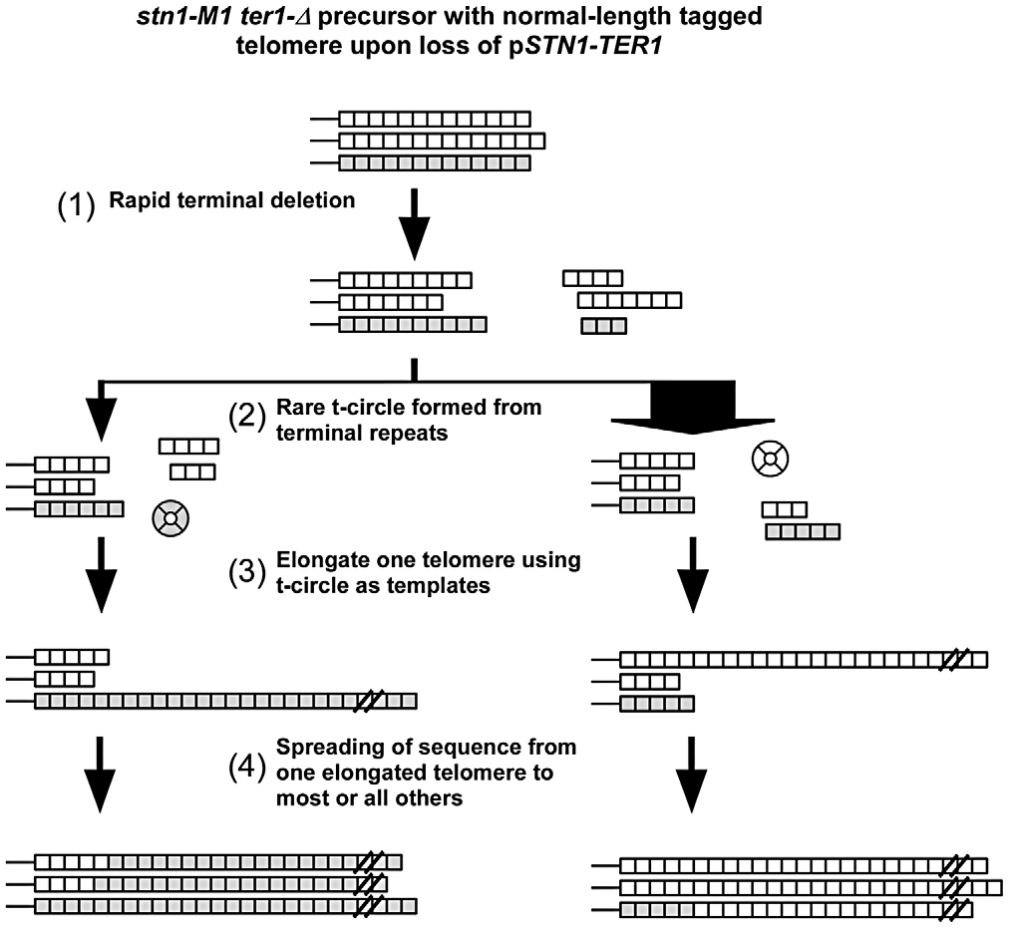
Modified roll-and-spread model of recombinational telomere elongation during the establishment of *stn1-M1 ter1-Δ* cells. (1) Upon loss of the complemented wild type *STN1* and *TER1* alleles, the terminal telomeric repeats are subject to rapid deletion; (2) A rare formation of a t-circle, likely as a deletion product of a single telomere. As 11 of 12 telomeres are composed of wild type repeats, most t-circles formed would have wild type repeats, as indicated by the width of the arrows; (3) At least one telomere is elongated through rolling circle synthesis using the t-circle as the template; (4) the sequence of the elongated telomere is spread to most or all other telomeres.

Taken together, our results suggest that the establishment of long and heterogeneous telomeres in *stn1-M1* via type IIR RTE may involve the following events as summarized in Figure 7. First, upon initiating the *stn1-M1* state, the uncapped telomeres rapidly undergo net deletion to generally lose at least one third of the repeats from telomeric termini. Next, an occasional telomeric recombination event results in the production of a small t-circle that is used as a template to lengthen one or more telomeres. These t-circles are likely derived from the more basal repeats of a telomere as indicated by the absence of amplification of ApaL repeats in most clones in our experiments. Finally, the initial elongated telomere(s) serve as the templates for lengthening most or all remaining telomeres, generally before other t-circles can form and compete for being copied. Another study of ours [48] that examined the stability of tandem arrays at telomeres in *stn1-M1* cells, suggests that once the long telomere state is established, the maintenance of it likely includes secondary formation and copying of t-circles. However, the spreading of sequence from a single source to all telomeres in these secondary amplifications was rare or absent, presumably because cells already contained many potential templates (both t-circles and linear telomeric tracts) that could be copied to generate long telomeric arrays.

The extent to which our results with *stn1-M1* cells bear on human ALT cells remains to be determined. T-circles are known to be abundant in ALT cells [17], however, there are conflicting data regarding their importance. While Ku mutations inhibit formation of t-circles and block proliferation of ALT cells [71], mutations in Xrcc3 and Nbs1 have been reported to eliminate t-circles without blocking ALT cell growth [72]. Our study suggests that it will be important to examine not only the maintenance of long telomeres in established ALT cells but also the role of t-circles during the initial establishment of the ALT cell state. Our data also suggest that a single telomeric template DNA might commonly be the source of elongation of multiple, even all, other telomeres. Interestingly, multiple telomeres in ALT cells have been shown to cluster into single foci which co-localize with PML bodies in a manner that could potentially facilitate recombination between telomeres [73]. However, there are other reasons to believe that a single telomere is unlikely to be the source of all telomere elongation during the establishment of ALT. These include that there far more telomeres in a mammalian cell and that the establishment of the ALT state might not be nearly as abrupt as the establishment of the *stn1-M1* state. Our finding that telomerase can have some effects on telomeric recombination in *stn1-M1* cells may have parallels in ALT. Indeed, expression of telomerase was shown to reduce the number of telomeres clustered in single foci in the ALT cells and could therefore decrease the frequency of recombination between telomeres [73]. Finally we would note that ALT does not arise in human cells from a simple lack of telomerase activity. This suggests that ALT likely requires mutations that allow telomeric recombination to occur at elevated frequencies. Mutations in yeast such as the *stn1-M1* mutation may suggest that alterations in human telomere binding proteins might be a possible cause or contributor to the ALT phenotype. Much further work will be needed to fully understand the mechanisms involved in the establishment and maintenance of ALT.

## Materials and Methods

### Strains and plasmid

All *K. lactis* strains used are derivatives of wild type (WT) 7B520 [74]. *K. lactis stn1-M1*, *stn1-M1 ter1-Δ* strains were described previously [42]. The precursors of *K. lactis stn1-M1 msh2-Δ* and *stn1-M1 ter1-Δ msh2-Δ* were constructed as follows. The *MSH2* gene was first amplified as a 4.4 kb fragment from genomic DNA of 7B520 cells by PCR (forward primer: 5′AGGGATCCGGGAGGCTCCAATAACAACA3′; reverse primer: 5′ACCTCGAGTTGCGAGTGATTCGTTCAAG3′) and cloned into the *Bam*HI and *Xho*I sites of pBluescript IIKS(−) resulting in p*MSH2*. Then, p*MSH2* was digested by *Bgl*II and *Eco*RI to delete an 891 bp fragment of the ORF of *MSH2,* and a 1.4 kb PCR-amplified (forward primer: 5′AGGCAGATCTGGATGGCGGCGTTAGTATCG3′; reverse primer: 5′AGGAATTCCCAGCGACATGGAGGCCCAG3′) fragment of *KANMX* gene from the genomic DNA of SAY557 [75] was inserted into the *Bgl*II+*Eco*RI− digested p*MSH2* to produce the p*MSH2*::*KANMX*. The 3.2 kb disruption cassette containing *MSH2*::*KANMX* was then amplified from p*MSH2*::*KANMX* by PCR (forward primer: 5′ATATTGCAGAGGAGCGAGGA3′; reverse primer: 5′CTTGTACGGACGGGTCATCT3′) and was transformed into either *stn1-M1* cells complemented with p*STN1* or *stn1-M1 ter1-Δ* cells complemented with p*STN1*-*TER1* or p*STN1*-*TER1(ApaL)*. The knockout of the *MSH2* gene was confirmed by Southern blotting and hybridization to a *MSH2* probe.

The plasmid p*STN1* was constructed in following two steps. First, a 3.4 kb fragment containing the 1.3 kb ORF of the *STN1* gene and 1.6 kb upstream and 0.5 kb downstream sequences was obtained by PCR (forward primer: 5′ACGAGCTCTGGCAACCCACTTGTGACTA3′; reverse primer: 5′ACCTCGAGTGCTCAGCCAATTTCTGTTG3′) using the genomic DNA of the 7B520 strain as the template. Second, the PCR fragment, which contains flanking *Sac*I and *Xho*I sites, was inserted into the polylinker *Sac*I and *Xho*I sites of pKL313(*HIS3*) [51] to generate the p*STN1*. Plasmids p*STN1*-*TER1* and p*STN1*-*TER1(ApaL)* were constructed as follows. First, the *STN1* gene was cloned as a 3.4 kb fragment from genomic DNA of 7B520 cells by PCR (forward primer: 5′ACGAGCTCTGGCAACCCACTTGTGACTA3′; reverse primer: 5′ACGGTACCTGCTCAGCCAATTTCTGTTG3′) into the *Sac*I and *Kpn*I sites of pCXJ18 [76] to result in plasmid pCXJ18-*STN1*. The 2.6 kb *TER1* or *TER1(ApaL)* gene fragments flanked by *Xba*I and *Kpn*I sites were obtained from pJR31 [37], and pJR31 derivative that contained a mutation in the template of *TER1* changing T20 to C to create an *Apa*LI restriction site by oligonucleotide mediated site-directed mutagenesis as described elsewhere [77]. Subsequently, the 2.6 kb *TER1* or *TER1(ApaL)* fragment was cloned into the pCXJ18-*STN1* to generate p*STN1*-*TER1* or p*STN1*-*TER1(ApaL)*.

The *URA3*-tagged single Bcl telomeres containing ∼11 and ∼40 Bcl-telomeric repeats were described before [37]. The *HIS3*-tagged single Bcl telomeres were based on the *URA3*-tagged single Bcl telomeres, which were cleaved by *Pst*I and *Nru*I to excise the *URA3* gene and replace it with a 1.2 kb *HIS3* fragment amplified from pKL313 [51] by PCR (forward primer: 5′ACAGTGCTGCAGCGGCATCAGAGCAGATTGTA3′; reverse primer: 5′ACTGAGTCGCGATCTGTGCGGTATTTCACACC3′). Either *URA3*-tagged or *HIS3*-tagged single Bcl telomeres were transformed into *stn1-M1* cells with a *MSH2* or a *msh2-Δ* genetic background complemented by p*STN1*, or *stn1-M1 ter1-Δ* cells with a *MSH2* or a *msh2-Δ* genetic background complemented by p*STN1*-*TER1* respectively. Ura^+^ or His^+^ colonies were then examined by Southern blotting to confirm that *URA3* or *HIS3*-tagged single Bcl telomeres had replaced a single native telomere by subtelomeric recombination.

*K. lactis* transformation was done by electroporation as described for *S. cerevisiae* [78]. Passaging of complemented cells was carried out by serial streaking of single colonies on rich medium (YPD plates) at 30°C. Strains were streaked every 3 days down to single cells that grew into colonies. Each streak was estimated to be 20–25 cell divisions.

### Telomere cloning and sequencing

The yeast genomic DNA sample from clone A1 in Figure 2A was partially digested with *Apa*LI. This was terminated by an equal volume of 12.5 mM EDTA added to the digestion reaction. The *Apa*LI partially digested DNA was ligated with the *Apa*LI-digested pACYC177 plasmid, and transformed into DH5α cells. The clones with telomeric fragments were confirmed by a Southern blot hybridized to telomeric probe. Positive clones were then sequenced.

### Southern hybridizations

Yeast genomic DNA samples digested with restriction enzymes were run on 0.8% or 3% agarose gels and then transferred onto Hybond N+ membrane in 0.4 M NaOH. All hybridization were carried out in Na_2_HPO_4_ and SDS as described [79]. The γ-^32^P-labeled telomeric probe is Klac 1–25 [63] The temperature of hybridization and washing for this probe was between 45–50°C. The γ-^32^P-labeled Bcl telomeric probe was KTelBcl (GATCAGGTATGTGG) [51] The temperature of hybridization and washing was 40°C and 34–36°C respectively. The subtelomeric probe was generated from pKL11-B (Insert of ∼1 kb telomeric *Eco*RI-*Sma*I fragment into pBluescript SK−), which was digested with *Xba*I and ligated back together to excise all the telomeric sequence and was then digested by *Eco*RI and *Xba*I to generate a ∼600 bp subtelomeric fragment for probe. The *URA3* probe was described before [80]. The *RAD50* gene probe was the purified PCR product from *K. lactis* genomic DNA (Forward primer: 5′GATAGGTCTACCGCGACCAA3′; Reverse primer: 5′GCGTAAGAGGACGCATTCAT3′). Subtelomeric, *URA3*, and *RAD50* probes were prepared using a random priming kit (NEB). Temperature of hybridization and washing for these probes was 65°C. The membranes were autoradiographed and visualized using a Molecular Dynamics Storm PhosphorImager.

## Supporting Information

Figure S1 The structure of two telomeres cloned from ApaL precursors. (A) Sequences of two cloned telomeres from ApaL precursors. The sequences of WT repeats (denoted as WT), ApaL repeats (denoted as ApaL) and half WT repeats (denoted as 1/2WT) are shown. The point mutation in the ApaL repeat is denoted with the asterisk. The number of repeats is shown in the subscript. (B) A potential mechanism that could generate the half WT repeat. The top panel shows the *K. lactis* telomerase RNA template region (bottom strand) with the imperfect 9 nt terminal repeats (arrows) that function during the translocation step of telomeric repeat synthesis [81]. The top strand indicates the 25 nt telomeric repeat expected to be synthesized. While the normal translocation of telomerase (middle panel) leads to the synthesis of a whole repeat, an abnormal translocation of telomerase (lower panel) due to a particular misalignment between the template and telomeric DNA could generate the observed half WT repeat. The sequence of the repeat that could be synthesized in each case is shown underlined. Drawings depict synthesis stopping before the last nt of the template, consistent with in vitro data [82].
(TIF)

Figure S2 Sequences of telomeric fragments cloned from the A1 clone. Shown are diagrams of 38 telomeric fragments cloned from *Apa*LI partially digested DNA from the A1 *stn1-M1 ter1-Δ* clone derived from an ApaL precursor strain. The sequences and names of different boxes illustrated in the patterns are shown. The number of clones recovered with the same DNA sequence is indicated at left.
(TIF)

Figure S3 Spreading of a telomeric sequence form a single telomere source during RTE in *stn1-M1 TER1* cells. (A) Southern blot, hybridized with a telomere probe, of *Eco*RI (indicated by “−”) and *Eco*RI+*Bcl*I (indicated by “+”) digested DNA from nine newly generated *stn1-M1 TER1 MSH2* clones from normal Bcl precursors in a *TER1 MSH2* background as well as one normal length Bcl *TER1 MSH2* precursor. (B–D) Same filter as in panel A after stripping and rehybridization with a Bcl telomere probe, subtelomeric probe and *URA3* probe, respectively. (E) Southern blot, hybridized with a telomere probe, of *Eco*RI (indicated by “−”) and *Eco*RI+*Bcl*I (indicated by “+”) digested DNA from 10 newly generated *stn1-M1 TER1 MSH2* clones from long Bcl precursors in a *TER1 MSH2* background. (F–H) Same filter as in panel E after stripping and rehybridization with a Bcl telomere probe, subtelomeric probe and *URA3* probe respectively. (I) Southern blot, hybridized with a telomere probe, of *Eco*RI (indicated by “−”) and *Eco*RI+*Bcl*I (indicated by “+”) digested DNA from seven newly generated *stn1-M1 TER1 msh2-Δ* clones from normal Bcl precursors in a *TER1 msh2-Δ* background as well as 2 normal length Bcl *TER1 msh2-Δ* precursor controls and an *stn1-M1* mutant (M1) control. (J–L) Same filter as in panel I after stripping and rehybridization with a Bcl telomere probe, subtelomeric probe and *URA3* probe respectively.
(TIF)
