# Supplementary figures and images for: Long Telomeres Produced by Telomerase-Resistant Recombination Are Established from a Single Source and Are Subject to Extreme Sequence Scrambling
Source: PLoS Genet. 2012 Nov 1;8(11):e1003017. doi: 10.1371/journal.pgen.1003017 (PMC3486848; doi:10.1371/journal.pgen.1003017)

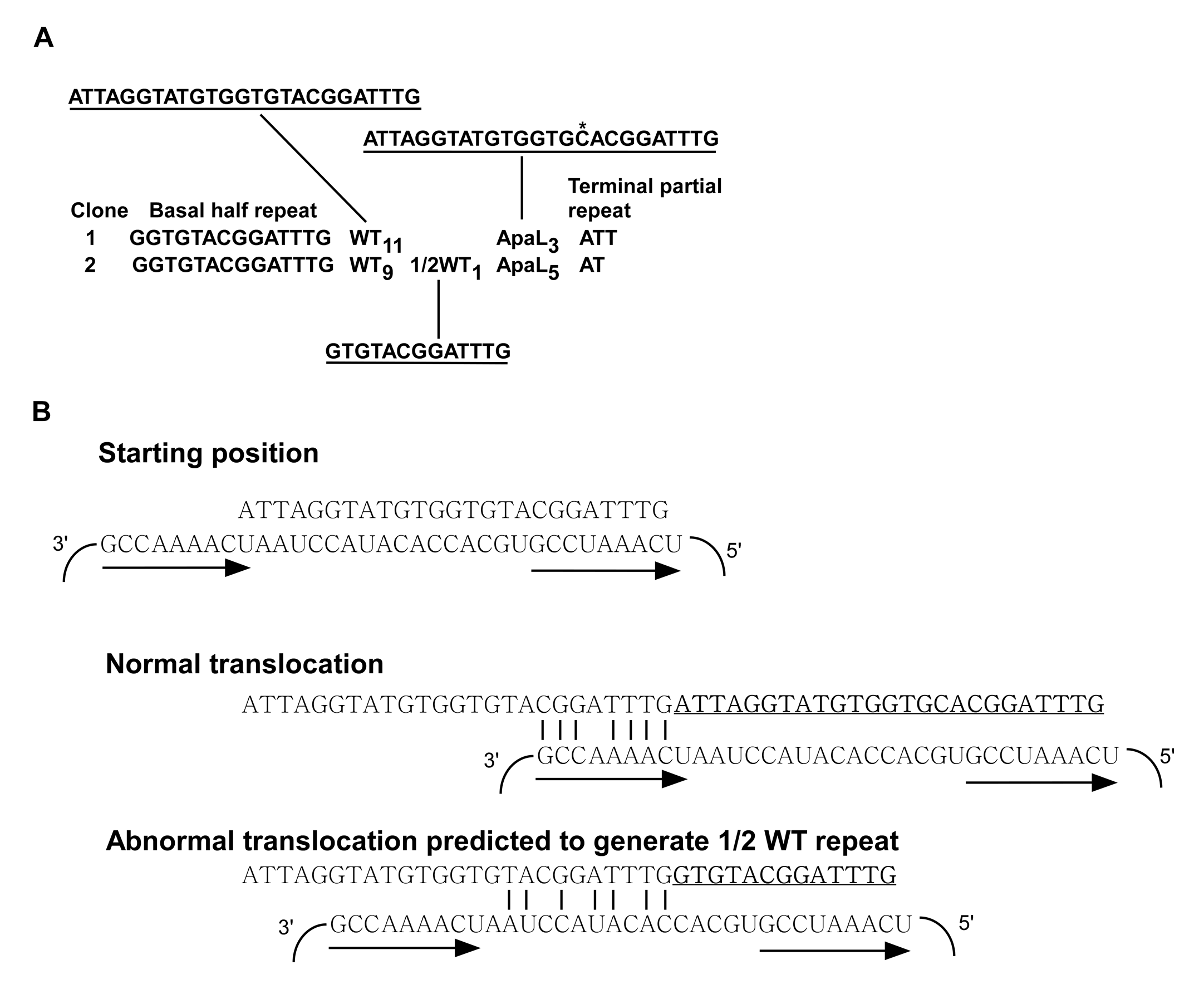

Supplement: Figure S1 — The structure of two telomeres cloned from ApaL precursors. (A) Sequences of two cloned telomeres from ApaL precursors. The sequences of WT repeats (denoted as WT), ApaL repeats (denoted as ApaL) and half WT repeats (denoted as 1/2WT) are shown. The point mutation in the ApaL repeat is denoted with the asterisk. The number of repeats is shown in the subscript. (B) A potential mechanism that could generate the half WT repeat. The top panel shows the K. lactis telomerase RNA template region (bottom strand) with the imperfect 9 nt terminal repeats (arrows) that function during the translocation step of telomeric repeat synthesis [81]. The top strand indicates the 25 nt telomeric repeat expected to be synthesized. While the normal translocation of telomerase (middle panel) leads to the synthesis of a whole repeat, an abnormal translocation of telomerase (lower panel) due to a particular misalignment between the template and telomeric DNA could generate the observed half WT repeat. The sequence of the repeat that could be synthesized in each case is shown underlined. Drawings depict synthesis stopping before the last nt of the template, consistent with in vitro data [82]. (TIF) [file pgen.1003017.s001.tif]

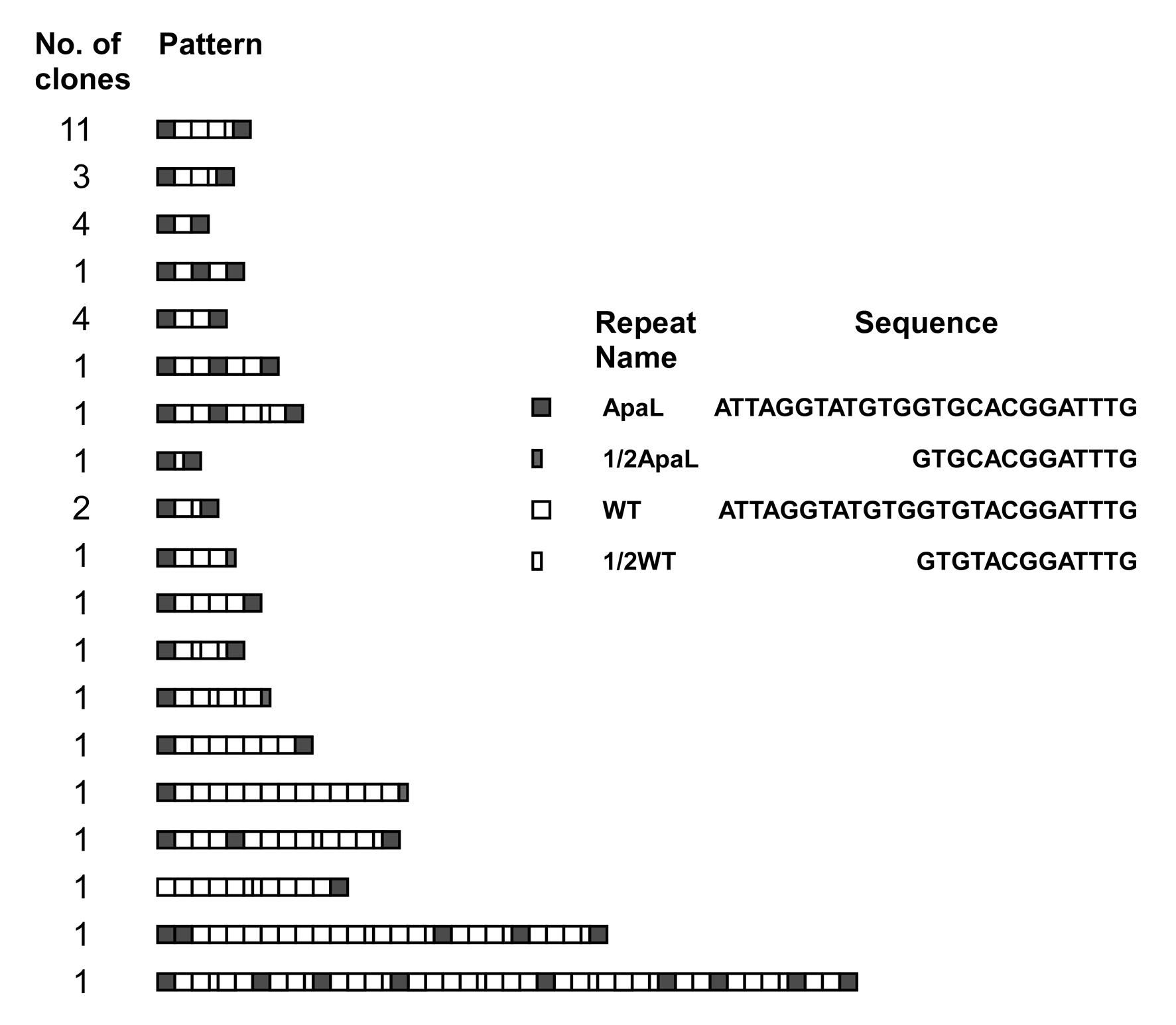

Supplement: Figure S2 — Sequences of telomeric fragments cloned from the A1 clone. Shown are diagrams of 38 telomeric fragments cloned from ApaLI partially digested DNA from the A1 stn1-M1 ter1-Δ clone derived from an ApaL precursor strain. The sequences and names of different boxes illustrated in the patterns are shown. The number of clones recovered with the same DNA sequence is indicated at left. (TIF) [file pgen.1003017.s002.tif]

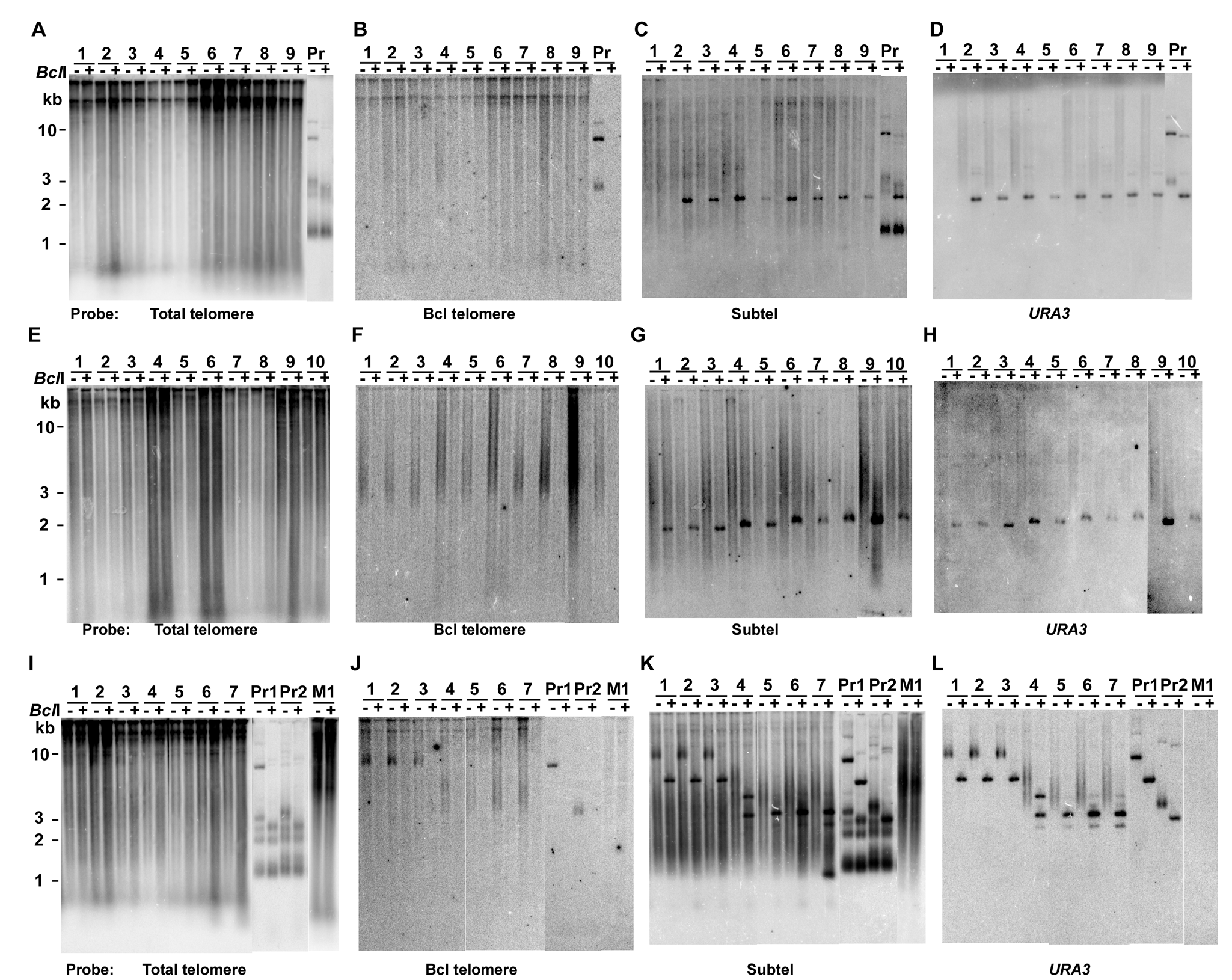

Supplement: Figure S3 — Spreading of a telomeric sequence form a single telomere source during RTE in stn1-M1 TER1 cells. (A) Southern blot, hybridized with a telomere probe, of EcoRI (indicated by “−”) and EcoRI+BclI (indicated by “+”) digested DNA from nine newly generated stn1-M1 TER1 MSH2 clones from normal Bcl precursors in a TER1 MSH2 background as well as one normal length Bcl TER1 MSH2 precursor. (B–D) Same filter as in panel A after stripping and rehybridization with a Bcl telomere probe, subtelomeric probe and URA3 probe, respectively. (E) Southern blot, hybridized with a telomere probe, of EcoRI (indicated by “−”) and EcoRI+BclI (indicated by “+”) digested DNA from 10 newly generated stn1-M1 TER1 MSH2 clones from long Bcl precursors in a TER1 MSH2 background. (F–H) Same filter as in panel E after stripping and rehybridization with a Bcl telomere probe, subtelomeric probe and URA3 probe respectively. (I) Southern blot, hybridized with a telomere probe, of EcoRI (indicated by “−”) and EcoRI+BclI (indicated by “+”) digested DNA from seven newly generated stn1-M1 TER1 msh2-Δ clones from normal Bcl precursors in a TER1 msh2-Δ background as well as 2 normal length Bcl TER1 msh2-Δ precursor controls and an stn1-M1 mutant (M1) control. (J–L) Same filter as in panel I after stripping and rehybridization with a Bcl telomere probe, subtelomeric probe and URA3 probe respectively. (TIF) [file pgen.1003017.s003.tif]
